# Physicochemical Properties, Thermal Stability, and Pyrolysis Behavior of Antioxidative Lignin from Water Chestnut Shell Obtained with Ternary Deep Eutectic Solvents

**DOI:** 10.3390/molecules28104088

**Published:** 2023-05-15

**Authors:** Feng Li, Wenzhi Lv, Dena Huang, Chenglu Zeng, Runping Wang

**Affiliations:** Ethnic Medicinal Plant Resources Development Engineering Research Center of Guizhou, School of Chemistry and Chemical Engineering, Qiannan Normal University for Nationalities, Duyun 558000, China; huagonglflf@163.com (F.L.); lvwenzhi@sgmtu.edu.cn (W.L.); huangdena1211@163.com (D.H.); zengcl@sgmtu.edu.cn (C.Z.)

**Keywords:** deep eutectic solvent, lignin, water chestnut shell, antioxidant activity, pyrolysis

## Abstract

The molecular weight of lignin extracted from lignocellulosic biomass is an important factor in determining its valorization in industrial processes. Herein, this work aims to explore the extraction of high molecular weight and bioactive lignin from water chestnut shells under mild conditions. Five kinds of deep eutectic solvents were prepared and applied to isolate lignin from water chestnut shells. The extracted lignin was further characterized with element analysis, gel permeation chromatography, and Ultraviolet-visible and Fourier-transform infrared spectroscopy. The distribution of pyrolysis products was identified and quantified with thermogravimetric analysis—Fourier-transform infrared spectroscopy and pyrolysis-gas chromatograph-mass spectrometry. The results showed that choline chloride/ethylene glycol/*p*-toluenesulfonic acid (1:1.8:0.2 molar ratio) exhibited the highest fractionation efficiency for lignin (84.17% yield) at 100 °C for 2 h. Simultaneously, the lignin showed high purity (90.4%), high relative molecular weight (37,077 g/mol), and excellent uniformity. Furthermore, the aromatic ring structure of lignin remained intact, consisting mainly of *p*-hydroxyphenyl, syringl, and guaiacyl subunits. The lignin generated a large number of volatile organic compounds during the depolymerization process, mainly composed of ketones, phenols, syringols, guaiacols, esters, and aromatic compounds. Finally, the antioxidant activity of the lignin sample was evaluated with the 1,1-diphenyl-2-picrylhydrazyl radical scavenging assay; the lignin from water chestnut shells showed excellent antioxidant activity. These findings confirm that lignin from water chestnut shells has a broad application prospect in valuable chemicals, biofuels and bio-functional materials.

## 1. Introduction

The water chestnut is widely cultivated in several provinces in China. Water chestnut shells (WCSs), as the main by-product in water chestnut production, are mostly incinerated or discarded, resulting in environmental pollution and resource waste. Polysaccharides and polyphenols contained in WCSs have been developed and applied in the pharmaceutical industry, but their high lignin content has been neglected. Furthermore, the natural structural properties and high-value applications of lignin obtained from WCSs have not been fully investigated. Lignin is an aromatic polymer composed of a variety of chemical bonds linked to phenylpropanol derivatives, containing a variety of reactive functional groups, which can be converted into fuels, chemicals, and bio-functional materials [1]. The key to the high-value utilization of lignin is to develop efficient separation methods. Currently, lignin is commonly extracted from lignocellulosic biomass using acids, alkalis, and organic solvents, but these methods operate under harsh conditions, causing environmental problems [2]. Therefore, it is urgent to find an environmentally friendly separation method to develop the lignocellulose refining industry. Recently, many environmentally clean technologies, such as green solvent and supercritical fluid extractions, have been reported for lignin extraction [3,4]. However, these techniques have various drawbacks. For example, the separation of lignin using ionic liquid has attracted attention due to the advantages of a mild separation process and high efficiency, but its high cost and the lack of toxicological data limit its industrial applications [3]. With the discovery of low-cost and easy-to-prepare deep eutectic solvents (DESs) for lignin fractionation [5], the development of green, efficient, and highly selective DES systems has become a research hotspot.

DES is a liquid eutectic mixture formed by mixing hydrogen bond donors (HBDs) and hydrogen bond acceptors (HBAs) at a certain molar ratio [6]. According to pH, DESs are divided into three categories, namely neutral, acidic, and alkaline [7]. Recent studies have demonstrated the strong advantages of acidic DESs for lignin separation. For example, TAN et al. compared the ability of neutral, acidic, and alkaline DESs to separate lignin from empty fruit bunch of oil palm [8,9]. Among those, the yield of lignin extracted with acidic DESs was significantly higher than that of the other two types, while the yield of lignin extracted with short-chain alkyl acid DESs was higher than that of linear saturated acid DESs [8,9]. In addition, Zhang et al. synthesized DESs for separating lignin from corncob using choline chloride with monocarboxylic acid, dicarboxylic acid, or polyol [10]. The lignin extraction yield was closely related to the chemical properties and acidity of the hydrogen bond donors [10,11]. Although carboxylic acid-based DESs can efficiently separate lignin, the β-*O*-4 and C–C bonds in the original lignin are destroyed during the treatment, resulting in low molecular weight and condensation reactions; these are not conducive to the high-value utilization of lignin in later stage [12].

The molecular weight of lignin directly affects its physicochemical properties. Lignin with low molecular weight and high hydroxyl content is suitable for the preparation of bioactive materials [13], while lignin with high molecular weight is an ideal raw material for the preparation of carbon fibers and other materials due to its high carbon content and high viscosity [14]. Lignin obtained using different methods varies greatly, mainly in terms of chemical structure and molecular weight. Generally, lignin separated with DES has a low molecular weight, usually in the range of 1000–6000 g/mol [15,16], probably due to the large number of ruptures of β-*O*-4 in the lignin structure. So far, the ball milling and γ-valerolactone (GVL)/H_2_O solvent methods can preserve the natural lignin structure of lignocellulose biomass, but the extraction efficiency is low [17,18]. Although the separation of lignocellulose with DES can improve the yield of lignin extracted at high temperatures or for longer periods, most of the β-*O*-4 bonds in the lignin structure are depolymerized during the process [19]. Provost et al. found that the lignin was extracted under mild conditions from softwoods using lactic acid-based DES, with an extraction yield of 78% and β-*O*-4 ether linkage retention of 61% [20]. To maximize the value of lignin, no matter which method is used for separation, serious changes in the chemical structure of lignin should be avoided during the extraction.

Based on previous research, lignin separated using carboxylic acid or alcohol-based DESs can retain a large number of β-*O*-4 bonds [21]. In addition, *p*-toluenesulfonic acid as a solvent showed good lignin removal ability under mild conditions, and the recovered lignin had high purity and moderate molecular weight [22,23]. To our knowledge, there have been few reports on the recovery of lignin from WCS using DES. In this study, a series of DESs were designed and synthesized to separate lignin from WCSs using choline chloride (ChCl) as hydrogen bond acceptor and ethylene glycol (EG), glycerol (GL), lactic acid (LA), oxalic acid (OA), and *p*-toluenesulfonic acid (*p*-TsOH) as hydrogen bond donors. The separated lignin was characterized using an elemental analyzer, gel permeation chromatography (GPC), Ultraviolet-visible (UV-Vis) spectroscopy, Fourier-transform infrared spectroscopy (FTIR), thermogravimetric analysis (TG)-FTIR, and pyrolysis-gas chromatograph-mass (Py-GC/MS) spectrometry. Its antioxidant activity was evaluated using the 1,1-diphenyl-2-picrylhydrazyl (DPPH) radical scavenging assay. This study is expected to provide useful references for the high-value utilization of water chestnut processing waste and the development of bioactive functional materials.

## 2. Results and Discussion

### 2.1. Yield, Molecular Weight Distribution, and Antioxidant Activity of Lignin Extracted with Different Type DESs

This study focused on obtaining lignin with high purity and high bioactivity for the future development of lignin biorefined chemicals. Herein, lignin was extracted from WCSs using five ChCl-based DESs under mild conditions. The results are illustrated in Table 1. Under the same conditions, carboxylic acid-based DESs had a better lignin extraction yield than alcohol-based DESs, with the highest yield of 84.17% for choline chloride/ethylene glycol/*p*-toluenesulfonic acid (ChCl/EG/*p*-TsOH). This result may be due to the presence of active protons in an acidic environment, which may break the ether bonds between the lignin and carbohydrate complexes, thus making the lignin more soluble. However, alcohol-based DESs lack active protons and acidic sites [24]. Additionally, ChCl/EG/*p*-TsOH has a relatively low viscosity among DESs. Many studies have demonstrated that low-viscosity DESs penetrate more readily into plant cells to remove lignin and hemicellulose [25]. The purity of lignin isolated using three acidic DESs was higher than 90%, with the highest obtained using choline chloride/oxalic acid (ChCl/OA), due to the strong hydrogen bond acceptability and high acidity of chloride salts in acidic DESs.

Further research on the lignin separated using the five kinds of DESs showed that the weight of lignin, i.e., the average molecular weight, varied greatly from 2884 to 37,077 g/mol. Those were related to the degree of lignin condensation and the β-*O*-4 content. The average molecular weight of the lignin isolated using ChCl/OA was lower than 2900 g/mol, probably due to the disruption of ether bonds in the structure of the lignin during extraction with DES, thus decreasing its molecular weight. However, the average molecular weight of the lignin separated using the DESs containing ethylene glycol was higher than 11,000 g/mol, while that of the lignin extracted using ChCl/EG/*p*-TsOH was the highest at 37,077 g/mol. This result may be because the main connecting bonds (ether bonds) in the lignin were not destroyed, thus preserving its original structure. Previous studies reported that the addition of ethylene glycol to DES can effectively preserve the β-*O*-4 bond in lignin and inhibit lignin condensation [26,27]. Moreover, the polydispersity index (PDI) can directly reflect homogeneity in the molecular weight distribution of lignin; when the PDI is lower than 3, lignin is considered to be a homogeneous polymer [28]. Compared with the PDIs of the lignin isolated from WCSs using alkaline, acetic acid, and *p*-TsOH [29], those of the lignin separated using the five DESs were all lower than 2.1, indicating more uniform structure for the lignin obtained using the latter. Lignin with high molecular weight has a wide range of applications. According to related studies, lignin with high molecular weight is suitable for the preparation of carbon fiber materials [30].

**Figure 1:**
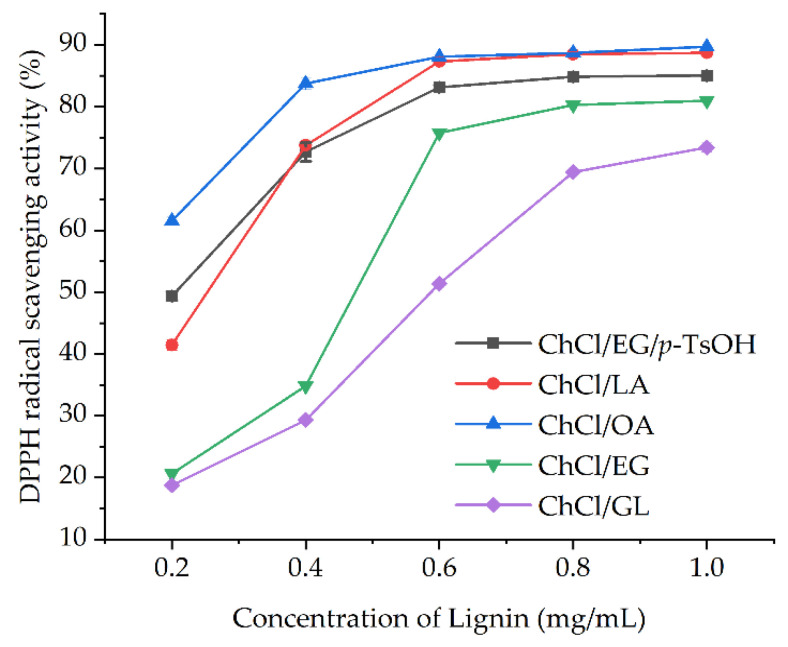
DPPH radical scavenging activity of the different types of DES-isolated lignin.

Lignin contains a large number of phenolic hydroxyl groups and has potential as a natural antioxidant. Many studies have found that the phenolic hydroxyl content in lignin has a direct relationship with the extraction method and the source of raw materials [31]. Therefore, the scavenging activity of the lignin extracted using the five DESs was investigated against DPPH radicals. As shown in Figure 1, the scavenging activity against DPPH radicals gradually increased with increasing lignin concentration and differed among the different types of lignin. The lignin isolated using carboxylic acid-based DES showed higher scavenging activity against DPPH radicals than the lignin obtained using alcohol-based DES. The highest scavenging activity against DPPH radical appeared at a 0.2 mg/mL concentration of the lignin obtained using ChCl/OA, followed by ChCl/EG/*p*-TsOH, choline chloride/lactic acid (ChCl/LA), and choline chloride/ethylene glycol (ChCl/EG). However, when the lignin concentration exceeded 0.6 mg/mL, the DPPH radical-scavenging activity of the lignin obtained using carboxylic acid-based DES increased slowly and remained mainly between 80% and 89%. When the lignin concentration was 1.0 mg/mL, the lignin obtained using the various DESs showed a scavenging activity of 73.41–89.73% against DPPH radicals, with ChCl/OA > ChCl/LA > ChCl/EG/*p*-TsOH > ChCl/EG > choline chloride/glycerol (ChCl/GL). The lignin separated using the five kinds of DESs showed different scavenging activity against DPPH radicals, mainly due to differences in the content of phenolic hydroxyl in the lignin structure. According to previous reports, lignin with a high content of phenolic hydroxyl possessed the best antioxidant ability [32,33].

In summary, under mild and short time conditions, lignin of high purity was obtained from WCSs with the separation method used in this study, without further purification in the subsequent process. The obtained lignin has good DPPH radical scavenging activity, indicating the potential to develop lignin-based functional materials. Since the lignin separated with ChCl/EG/*p*-TsOH DES had the highest molecular weight, the elemental composition, chemical structure, and pyrolysis products were further analyzed.

### 2.2. Elemental Composition Analysis of Lignin

Lignin composition determines its chemical structure and heating value. The C, H, O, S, and N contents in the lignin separated with ChCl/EG/*p*-TsOH DES were 52.81%, 3.76%, 41.43%, 0.03%, and 1.55%, respectively. The presence of small amounts of N and S is probably due to the presence of small amounts of protein. This result is similar to that of the reported lignin in sugarcane bagasse [33]. According to the mass fraction of C, H, and O and the relative atomic mass ratio, the empirical molecular formula for the lignin was C_9_H_7.69_O_5.29_ [34]. A higher heating value (HHV) is an important indicator to measure the maximum heating value of lignin. According to the Dulong heating formula described in the literature [35], the obtained lignin had an HHV of 15.85 MJ/kg. Compared with that from other reports, the lignin obtained from WCSs had a lower HHV. The same phenomenon was also found in the lignin (15.75 MJ/kg) from cashew apple bagasse obtained using acid–alkali pretreatment [2].

### 2.3. UV-Vis and FTIR Analysis of Lignin

**Figure 2:**
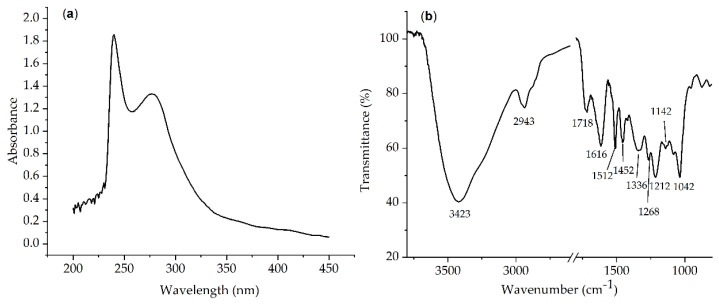
UV−Vis (**a**) and FTIR (**b**) spectra of the lignin isolated with ChCl/EG/*p*−TsOH DES.

Ultraviolet-visible spectroscopy is a simple and effective tool for the qualitative determination of the phenolic hydroxyl groups in lignin. With structures such as benzene rings, hydroxyl, methoxy groups, and carboxyl, lignin shows obvious ultraviolet absorption. Figure 2a shows the absorption spectrum of lignin in a 1,4-dioxane/H_2_O solution. Among those, two absorption peaks at 200–350 nm indicate that the substance is a phenolic compound. Usually, lignin has a typical absorption peak at both 240 nm and 280 nm due to the electron transfer from non-conjugated phenolic structures in aromatics [36].

Lignin has various types of functional groups, such as -OH, -OCH_3_, and -COOH; thus, its characteristic chemical structure can be intuitively reflected in FTIR spectra. The FTIR spectrum for the lignin isolated with ChCl/EG/*p*-TsOH is shown in Figure 2b. The FTIR absorption peaks in the lignin were assigned as reported in the literature [2,13,31,34]. The absorption peak at 3423 cm^−1^ was assigned to the stretching vibration in the aromatic and aliphatic hydroxyl groups. The absorption peak at 2943 cm^−1^ was attributed to the symmetric C–H stretch in the alkyl group. The absorption peak at 1718 cm^−1^ was attributed to the C=O stretching vibration in nonconjugated ketones and carbonyl groups, indicating that some benzene rings were destroyed during the extraction of lignin with DES, forming a quinoid structure. The absorption peak at 1616 cm^−1^ was assigned to the C=C conjugation stretch in the aromatic rings of coniferyl and sinapyl alcohols. The absorption peak at 1513 cm^−1^ was assigned to the vibration in the aromatic skeleton of lignin. The absorption peak at 1452 cm^−1^ was assigned to the asymmetric C–H deformation of methyl or methylene in the ether structure of guaiacyl, syringyl, and p-hydroxyphenylpropane (GSH)-type lignin. These absorption peaks confirmed that the benzene ring-based skeleton structure was well preserved during the extraction with DES. The absorption peak at 1336 cm^−1^ was assigned to the C–O groups corresponding to the syringl and guaiacol units. The peak at 1268 cm^−1^ was assigned to a guaiacyl unit and the C=O stretching vibration. The peak at 1212 cm^−1^ was ascribed to the C–O group. The peak at 1142 cm^−1^ corresponded to the aromatic ring of guaiacol and the C=O stretch. The peak at 1042 cm^−1^ corresponded to the C–H deformation of guaiacol, the C–O deformation in main alcohols, and the nonconjugated C=O stretch. Furthermore, there were no characteristic absorption peaks of carbohydrates at 1373 cm^−1^ and 1056 cm^−1^ [37]. The above results indicated that lignin with complete structure and high purity could be obtained by extraction with DES.

### 2.4. Thermal Analisis of Lignin

**Figure 3:**
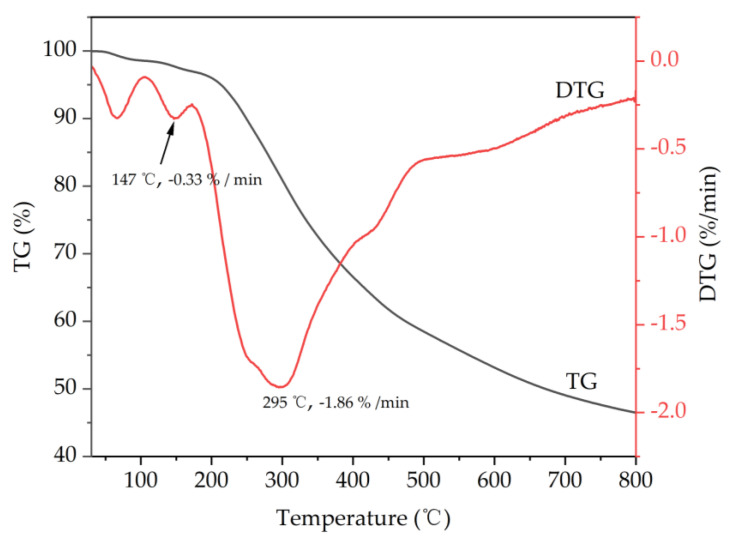
TG and DTG curves of the lignin isolated with ChCl/EG/*p*−TsOH DES.

Lignin is an organic polymer mainly composed of a phenylpropane unit and connected with various chemical bonds. The types of chemical bonds and functional groups directly affect the pyrolysis properties of lignin. To explore the thermal decomposition characteristics of the lignin extracted from WCSs with DES (ChCl/EG/*p*-TsOH), a thermal analysis was performed. The thermogravimetric (TG) and differential thermogravimetric (DTG) curves of the lignin are presented in Figure 3. According to the TG curves, the weight loss curve of the lignin was divided into three main stages, namely, the evaporation of free and bound water, depolymerization, and carbonization. Between 30 °C and 200 °C, the lignin lost approximately 4% of its mass at a weight loss rate of 0.33%/min. This stage was mainly due to the dehydration and volatilization of small molecule-based organic acids. The thermal decomposition rate of the lignin accelerated starting from 200 °C. At this stage, the thermal degradation of the lignin mainly involved chemical bond breaking (mainly C–O and C–C in the aryl ether structure) and side-chain oxidation (carbonylation, carboxylation, dehydrogenation, etc.) [38]. At temperatures above 400 °C, this stage mainly involved the cleavage of C–C bonds and demethoxylation of the benzene ring in the lignin skeleton structure [39], accompanied by the generation of coke. With the increase in temperature to 800 °C, the weight loss of the lignin slowed down gradually due to the condensation of the aromatic structure, resulting in a high residual amount of 46.49%.

TG curves may reveal lignin weight loss throughout various decomposition processes, while DTG curves can record the related maximum decomposition temperature (T_M_). Many studies have demonstrated that the T_M_ of lignin during the pyrolysis was proportional to the number of β-*O*-4 linkages present in the molecule [40]. According to the DTG curves, the maximum weight loss peak appeared at 295 °C, with a weight loss rate of 1.86%/min. However, the lignin extracted from WCSs with NaOH or *p*-TsOH showed the highest mass loss between 370 °C and 336 °C in our previous report [29]. The lignin extracted with ChCl/EG/*p*-TsOH-had a lower T_M_, indicating that there were more β-*O*-4 linkages in the lignin. Moreover, at higher temperatures (400 °C to 500 °C), the shoulder peak corresponded to the cleavage of the C–C bond in the lignin. It is inferred from the results of TG/DTG that the thermal behavior of lignin has an intrinsic relationship with its inherent structure.

**Figure 4:**
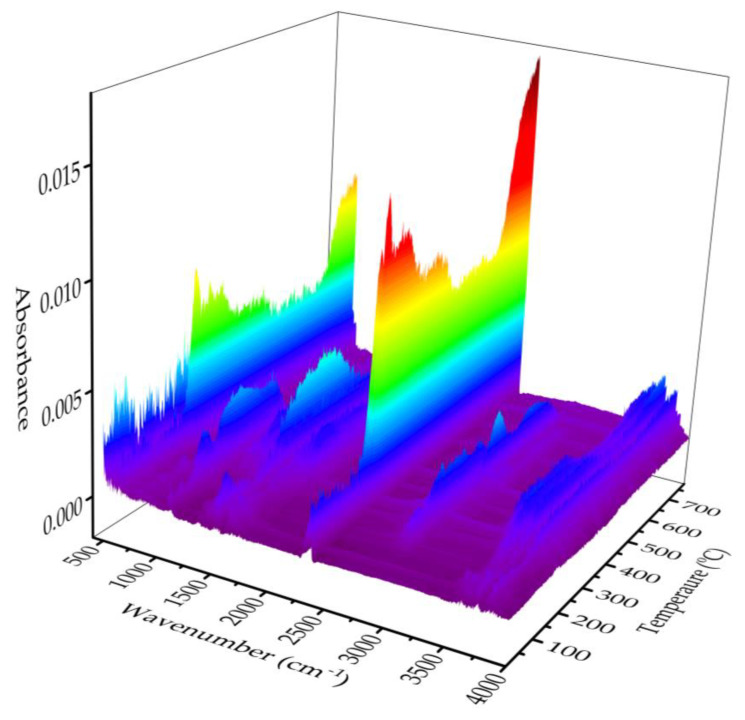
The 3D−FTIR spectra of ChCl/EG/*p*−TsOH−isolated lignin with a heating rate of 10 °C/min, where red, yellow, green, blue and purple represent very strong, strong, medium, weak and very weak infrared absorbance, respectively.

TG-FTIR spectroscopy is an effective tool for the dynamic monitoring of volatile substances. To further explore the types of volatile substances involved in the pyrolysis of lignin, 3D-FTIR spectra analysis was performed on the sample. As shown in Figure 4, the main thermal degradation of the lignin mainly occurred at 200–600 °C. A variety of volatile organic compounds were detected, with different absorption wavenumbers corresponding to their functional groups. The peak at 3500–3800 cm^−1^ corresponds to the H_2_O derived mainly from the dehydroxylation of the lignin. The peak at 2939 cm^−1^ corresponds to the CH_4_ derived mainly from demethoxy or methyl groups. As observed in other types of lignin [40], the maximum peak at 2310 cm^−1^ corresponds to CO_2_, a major pyrolysis product primarily derived from the decarboxylation of the lignin. The peak at 2185 cm^−1^ corresponds to the CO arising from the ether functional group in the side chain of the lignin, and only appears at high temperatures, without the obvious CO absorption peak at low temperatures. The multiple absorption peaks at 1800–1000 cm^−1^ were ascribed to many pyrolysis products such as alcohols, phenols, and aromatic compounds [39,40], resulting from the ring-opening reaction of the aromatic structure and the fragmentation in the side-chain of the lignin.

**Figure 5:**
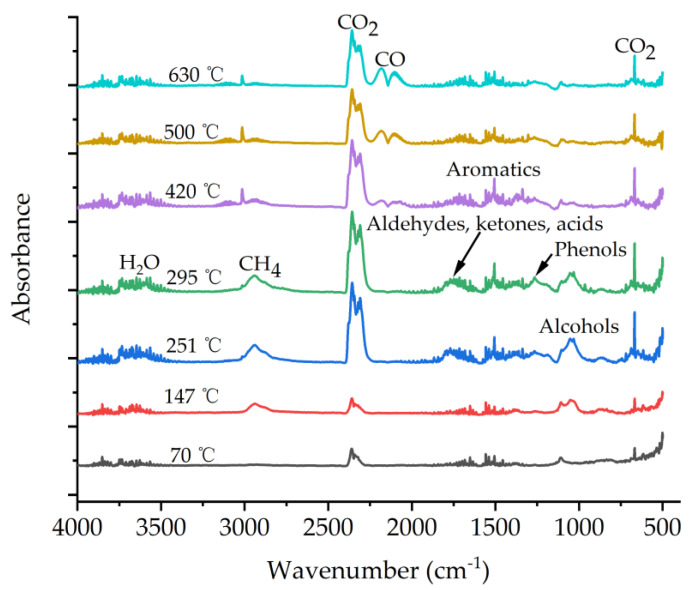
FTIR spectra of the volatile components in the vapor phase of the lignin isolated with ChCl/EG/*p*−TsOH DES.

Based on the results of TG and DTG, the change in volatiles with increasing temperature was also investigated. The FTIR spectra are depicted in Figure 5, involving volatile compounds during lignin pyrolysis at 70 °C, 147 °C, 251 °C, 295 °C, 420 °C, 500 °C, and 630 °C. The Lambert–Beer law states that the absorption intensity of a certain wavenumber was proportional to its concentration. Thus, changes in absorbance intensity throughout the pyrolysis may indicate a trend in product concentration. As shown in Figure 5, small amounts of volatiles, including H_2_O, CO_2_, CH_4_, and alcohols, were detected at low temperatures (70 °C and 147 °C); however, CH_4_ was not found at 70 °C. This result is also in accordance with the TG analysis shown in Figure 3, where the mass loss was approximately 4% (<200 °C). Moreover, it is worth noting that the increase in pyrolysis temperature led to a rise in the intensity of all absorption peaks. For example, at 251 °C and 295 °C, the concentration of volatiles began to increase, and new volatiles, such as aromatics, aldehydes, ketones, acids, and phenols, formed. This result is in line with that of the DTG analysis, where the maximum weight loss peak appeared at 295 °C. At higher temperatures (420 °C, 500 °C, and 630 °C), the absorption intensity of the volatiles showed a decreasing tendency with increasing pyrolysis temperatures, except for CO. This resulting trend suggests that thermochemical processes targeting the production of volatile products performed best at moderate temperatures between 250 °C and 300 °C. The TG-FTIR results indicate that lignin releases large amounts of organic matter, CO_2_, and CH_4_ during pyrolysis, with great potential to replace coal and natural gas as syngas feedstocks and raw materials for bioenergy production.

### 2.5. Py-GC/MS Analysis of the Depolymerized Products

**Figure 6:**
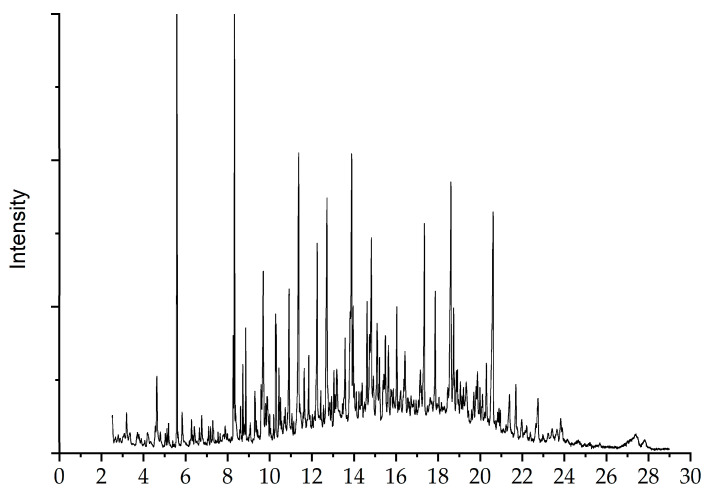
Total ion chromatograph of lignin extracted using ChCl/EG/*p*-TsOH.

The pyrolysis compounds, generated during the thermal degradation of the lignin, were separated using gas chromatography (GC) and identified using mass spectrometry (MS). The method facilitated an analysis of the structure and further exploration on the pyrolysis properties of the lignin, demonstrating the potential to transform lignin into high-value-added chemicals. The total ion chromatogram of the lignin is presented in Figure 6. The pyrolytic products and their peak area are summarized in Table 2. Thirty-four compounds (seen in Figure S1 in the Supplementary Materials) were identified according to the previously reported literature [41,42,43].

According to their structures, these pyrolysis compounds were classified into guaiacol (G) types, syringol (S) types, catechol (C) types, aromatics, ketones, and esters. The presence of GSH-type compounds in the pyrolysis products indicated that the lignin from WCSs belonged to the GSH type, further verifying the results of FTIR. Although it is impossible to obtain the exact yield of each chemical in the pyrolysis products, the peak area of the molecule in the chromatographic relates linearly to its relative content, which can be utilized to reveal a relative content change in each identified product. The peak area of lignin pyrolysis products follows the order of G-type > aromatic > S-type > esters > H-type > ketones > C-type compounds. The G-type compounds were the main pyrolysis products, derived from the G-type unit and probably resulting from the breakage of many β-*O*-4 bonds in the lignin. Similar results were previously mentioned by Zong et al. [44]. In addition, 2,4-Dimethylphenol and C-type compounds were found in lignin pyrolysis products, which may be due to the presence of condensed S units. Small amounts of aldehyde, ketones, and acids seemed to indicate the presence of carbonyl and carboxyl functional groups in the lignin structure. Furthermore, the type of lignin pyrolysis products was directly related to the pyrolysis temperature. According to a previous report, when the pyrolysis temperature of lignin was 600 °C, the content of phenolic compounds was higher, while that of alkoxyphenol compounds was lower [45]. At the pyrolysis temperature of 610 °C, the main pyrolysis products were phenols, toluene, and their corresponding derivatives. The peak area of methyl vanillin was the most abundant in all pyrolysis products. Those findings confirm that the lignin from WCSs has the potential to develop high-value-added chemicals, especially phenol, an important chemical currently produced mainly from petroleum feedstocks. In addition, in the future refining industry, lignin may be used as a raw material to prepare vanillin used in cosmetics, foods, and other fields.

## 3. Materials and Methods

### 3.1. Materials and Chemical Reagents

Water chestnut shells, from Shandong province, China, were collected and ground into 80-mesh particle samples after washing and air-drying. To remove ethanol and water-soluble extracts, the samples were extracted with benzene/ethanol (2:1 *v*/*v*) at 90 °C for 6 h in a Soxhlet extractor.

Choline chloride, ethylene glycol, *p*-toluenesulfonic acid monohydrate, oxalic acid dihydrate, lactic acid, glycerol, and other reagents (or solvents) were purchased from Aladdin Co., Shanghai, China. The used 2,2-Diphenyl-1-picrylhydrazyl (>98.5%) was purchased from Machlin Co., Shanghai, China.

### 3.2. Preparation of Deep Eutectic Solvent

The hydrogen bond acceptor (ChCl) and the hydrogen bond donors (EG, OA, LA, and *p*-TsOH) were mixed at different molar ratios and magnetically stirred at 80 °C until a transparent and homogeneous liquid was formed. The composition and corresponding molar ratios of the prepared DESs are summarized in Table 3 with their physical properties, such as viscosity and surface tension.

### 3.3. Lignin Extraction

**Figure 7:**
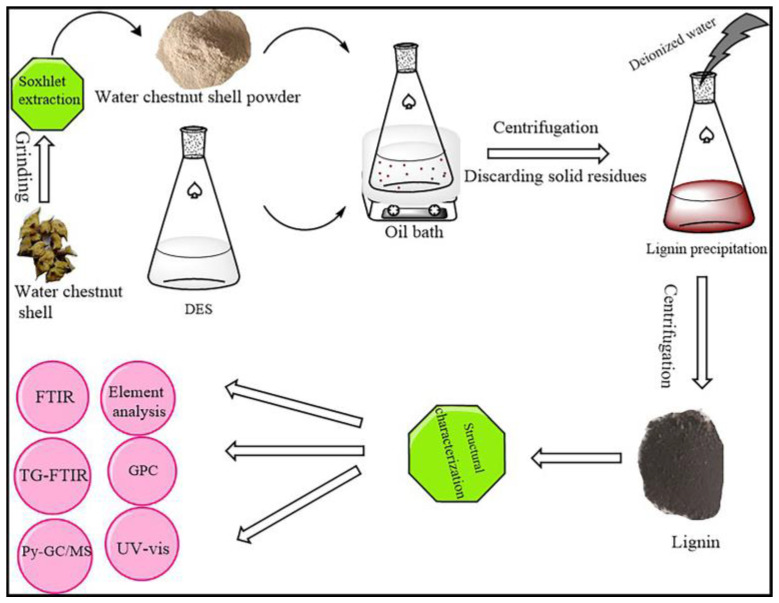
Schematic diagram showing lignin extraction with DES.

The lignin extraction procedure is shown in Figure 7. Using five kinds of DESs as solvents, 1 g of WCS powder was mixed with 20 mL of DES in a 200 mL round-bottom flask. The flask containing the mixture was placed in an oil bath at 100 °C, with continuous magnetic stirring for 2 h. Subsequently, the mixture was cooled to room temperature and centrifuged to obtain a transparent DES-based liquid phase. The solid residue was filtered using a vacuum and washed with anhydrous ethanol until the filtrate was clear and colorless. The resulting washing solution was combined with the DES liquid phase containing extracted lignin. The mixed solution was evaporated at 50 °C to remove ethanol. The concentrated solution was diluted with deionized water to precipitate lignin. The lignin was recovered with centrifugation and vacuum freeze-drying. According to the DES solvent used, the lignin obtained was labeled as ChCl/OA, ChCl/LA, ChCl/EG, ChCl/GL, and ChCl/EG/*p*-TsOH. The yield of lignin extracted from the WCSs was calculated using Equation (1),

(1) $$Y\left(\%\right)=\frac{m}{w}\times 100\%\;$$

where *m* denoted the lignin mass extracted from the WCSs and *w* denoted the total mass of lignin in the WCSs.

The contents of acid-soluble lignin (ASL) and acid-insoluble lignin (AIL) in biomass or lignin samples were determined according to the methods used in the previous literature [17,37]. Based on the previous literature, lignin purity was calculated from the total amount of ASL and AIL in lignin samples [15,46].

### 3.4. DPPH Radical Scavenging Activity Assay

The lignin extract of different masses was dissolved in a 1,4-dioxane aqueous solution to obtain several lignin solutions of 0.2–1.0 mg/mL. To a 10 mL brown centrifuge tube were added 0.1 mL of the lignin solution and 3.9 mL of a DPPH ethanol solution (25 mg/L), respectively. Each mixed solution was shaken and stood in a water bath at 25 °C for 30 min, with the absorbance measured at 517 nm with UV-Vis spectroscopy.

### 3.5. Analytical Procedures

#### 3.5.1. DES Characterization

The viscosity of the DESs was determined at 30 °C using a viscometer (Fungilab, Barcelona, Spain). The surface tension of the DESs was measured using a contact angle meter (Biolin Scientific Theta Flex, Gothenburg, Sweden).

#### 3.5.2. Molecular Weight and Elemental Analysis of Lignin

The elemental analysis (C, H, O, N, and S) of the lignin samples was performed using a Vario EL cube elemental analyzer (Elementar, Langenselbold, Germany). The relative molecular weight distribution of the lignin sample was measured with GPC according to the methodology proposed by Alvarez-Vasco et al. [14].

#### 3.5.3. UV-Vis and FTIR Spectroscopy Analysis of Lignin

The UV-Vis absorption spectra of the lignin were recorded on an ultraviolet-visible spectrophotometer (Persee, TU-1901, Beijing, China). About 5 mg of the lignin extract was dissolved in a 10 mL aqueous solution of 90% (v%) 1,4-dioxane. The absorbance was determined by scanning the solution over the wavelength range of 200–400 nm. Functional groups in the lignin were analyzed using FTIR (Thermo Fisher, Nicolet iS 20, Waltham, MA, USA). FTIR spectra were obtained in the range of 4000–400 cm^−1^ with a resolution of 4 cm^−1^ and 32 scans.

#### 3.5.4. TG-FTIR Analysis of Lignin

Thermal stability and volatiles generated during pyrolysis were carried out using a TG 209 F1 thermogravimetric analyzer (Netzsch, Selb, Germany) coupled with Nicolet iS 50 FTIR (Thermo Fisher, Waltham, MA, USA) at a temperature from 30 °C up to 800 °C at a heating rate of 10 °C/min under N_2_ atmosphere. Simultaneously, FTIR spectra of the volatiles were recorded in the region of 4000–400 cm^−1^.

#### 3.5.5. Py-GC/MS Analysis of the Depolymerized Products

The pyrolysis process was performed with a PY-2020Id pyrolyzer (Frontier Lab, Koriyama, Japan) at 610 °C for 30 s. The pyrolytic products present in the lignin samples were estimated using QP2010 Ultra GC/MS (Shimadzu, Kyoto, Japan) equipped with a UA-30M-0.25F capillary column (30 m × 0.25 mm × 0.25 μm) with high-purity helium as a carrier gas. The GC/MS operation parameters were an injector temperature of 300 °C, a helium flow of 1 mL/min, an increasing oven temperature from 50 °C to 280 °C with a rate of 8 °C/min, an electron ionization mode at 70 eV, and the m/z ranging from 30 to 550. The main pyrolytic products were identified using the NIST 11 library with a probability match (>80%) for the qualitative comparison of the products.

## 4. Conclusions

In this study, five kinds of DESs were synthesized, and the corresponding physicochemical properties were analyzed. Among these, the most effective DES was obtained by performing the isolation of lignin from the WCSs. Under mild conditions, the DES composed of ChCl/EG/*p*-TsOH could obtain a higher lignin extraction yield (84.17%). Simultaneously, the structure and composition of the extracted lignin were analyzed in detail using FTIR, TG-FTIR, and Py-GC/MS. The results demonstrated that the lignin separated with ChCl/EG/*p*-TsOH DES had the characteristics of high purity, high molecular weight (Mw = 37,077 g/mol), and low PDI (1.36) and relatively completely retained the original lignin structure. Furthermore, the pyrolysis characteristics of the lignin showed that it had good thermal stability and released a lot of volatile substances, such as ketones, phenols, syringols, guaiacols, esters, and aromatic compounds, during pyrolysis at high temperatures. When the lignin concentration was 1.0 mg/mL, the lignin obtained with various DESs showed a scavenging activity of 73.41–89.73% against DPPH radicals. The above results indicate that the lignin from WCSs has the potential as a high-value-added chemical.

## Figures and Tables

**Table 1:** Yield, purity, and molecular weight of lignin extracted with different methods.

Method	Yield (%)	Purity (%)	Mw (g/mol)	Mn (g/mol)	PDI	Reference
ChCl/OA	65.66	94.3	2884	1853	1.58	
ChCl/LA	55.62	92.7	4462	3095	1.44	
ChCl/EG	50.95	86.2	11,836	7019	1.68	This study
ChCl/GL	46.32	88.8	5871	2849	2.06	
ChCl/EG/*p*-TsOH	84.17	90.4	37,077	27,252	1.36	
*p*-TsOH	—	—	16,482	7640	2.15	[29]
NaOH	—	—	42,312	6813	6.21	[29]
Acetic acid	—	—	10,149	4140	2.45	[29]

Abbreviations: Mw, Weight average molecular weight; Mn, Number average molecular weight; PDI, Polydispersity index (Mw/Mn).

**Table 2:** The relative content of main pyrolytic products for lignin in Py-GC/MS.

R.T./min	Product Compounds	Peak Area (%)	Origin
4.63	Toluene	1	Aromatic
6.28	Ethylbenzol	0.26	Aromatic
6.42	1,3-Xylene	0.37	Aromatic
6.77	Phenethylene	0.42	Aromatic
8.36	Phenol	0.76	H-lignin
8.72	*p*-Methoxytoluene	0.86	Aromatic
9.29	2-methyl-Phenol	0.72	H-lignin
9.59	*p*-Methylphenol	0.91	H-lignin
9.68	Guaiacol	3.85	G-lignin
10.3	O, O-Dimethyl catechol	1.14	Catechol
10.43	2,4-Dimethylphenol	1.21	H-lignin
10.73	5-Methylguaiacol	0.21	G-lignin
11.37	3,4-Dimethoxytoluene	5.07	Aromatic
11.85	*p*-Ethylguaiacol	0.96	G-lignin
12.25	4-Ethyl-2-methoxyanisole	3.29	
12.72	3,4-Dimethoxystyrene	6.01	Aromatic
12.93	Cinnamic acid, methyl ester	0.45	
13.05	Eugenol methyl ether	0.74	G-lignin
13.19	Vanillic aldehyde	1.12	G-lignin
13.59	trans-Isoeugenol	1.75	G-lignin
13.89	Methylvanillin	8.19	G-lignin
13.96	Isoeugenyl methyl ether	1.88	G-lignin
14.01	Acetoguaiacon	0.64	G-lignin
14.23	Methyl vanillate	0.37	G-lignin
14.39	Guaiacylacetone	0.49	G-lignin
14.63	Ethanone, 1-(3,4-dimethoxyphenyl)	1.88	G-lignin
14.74	3,4-Dimethoxybenzyl methyl ketone	2.07	
14.82	Benzoic acid, 3,4-dimethoxy-, methyl ester	3.29	
15.22	1,2-Dimethoxy-4-(2-methoxyethenyl) benzene	1.19	S-lignin
15.5	1,2,4-trimethoxy-5-(1-propenyl)- Benzene	1.6	S-lignin
15.64	3,4-dimethoxy-Benzenepropanol	1.56	S-lignin
15.88	Benzenepropanoic acid, 3,4-dimethoxy-, methyl ester	0.22	
16.04	1,2-Dimethoxy-4-(3-methoxy-1-propenyl) benzene	1.94	S-lignin
17.16	Cinnamic acid, 3,4-dimethoxy-, methyl ester	0.73	

**Table 3:** The physical properties, composition, and corresponding molar ratios of DESs used in this work.

DES Components		Molar Ratio	Abbreviation	Viscosity (mPa·s)	Surface Tension (mN·m^−1^)
HBA	HBD				
ChCl	OA	1:2	ChCl/OA	ND	ND
ChCl	LA	1:2	ChCl/LA	145.1	40.37
ChCl	EG	1:2	ChCl/EG	35.54	49.74
ChCl	GL	1:2	ChCl/GL	503.4	49.92
ChCl	EG + *p*-TsOH	1:1.8:0.2	ChCl/EG/*p*-TsOH	39.4	47.83

ND: Not Detected.

## Data Availability

Not applicable.

## Supplementary Materials

The following supporting information can be downloaded at: https://www.mdpi.com/article/10.3390/molecules28104088/s1, Figure S1: GC/MS library hits for the lignin extracted using ChCl/EG/*p*-TsOH.

## Sample Availability

Not available.

## Abbreviations

WCS:	water chestnut shell
DES:	deep eutectic solvent
ChCl/EG/*p*-TsOH:	choline chloride/ethylene glycol/*p*-toluenesulfonic acid
ChCl/OA:	choline chloride/oxalic acid
ChCl/LA:	choline chloride/lactic acid
ChCl/EG:	choline chloride/ethylene glycol
DPPH:	1,1-diphenyl-2-picrylhydrazyl
Mw:	weight average molecular weight
Mn:	number average molecular weigh
PDI:	polydispersity index
