# Supplementary material for: Physicochemical Properties, Thermal Stability, and Pyrolysis Behavior of Antioxidative Lignin from Water Chestnut Shell Obtained with Ternary Deep Eutectic Solvents
Source: Molecules. 2023 May 15;28(10):4088. doi: 10.3390/molecules28104088 (PMC10223403; doi:10.3390/molecules28104088)
Supplement: Supplementary file 1 [file molecules-28-04088-s001.zip › molecules-2337960-supplementary.pdf]

molecules

MDPI

Supplementary Materials

Physicochemical Properties, Thermal Stability, and Pyrolysis Behavior of Antioxidative Lignin from Water Chestnut Shell Obtained with Ternary Deep Eutectic Solvents

Feng Li, Wenzhi Lv, Dena Huang, Chenglu Zeng and Runping Wang *

Ethnic Medicinal Plant Resources Development Engineering Research Center of Guizhou, School of Chemistry and Chemical Engineering, Qiannan Normal University for Nationalities, Duyun 558000, China; huagonglflf@163.com (F.L.); lvwenzhi@sgmtu.edu.cn (W.L.); huangdena1211@163.com (D.H.); zengcl@sgmtu.edu.cn (C.Z.)

* Correspondence: runpingw0826@163.com

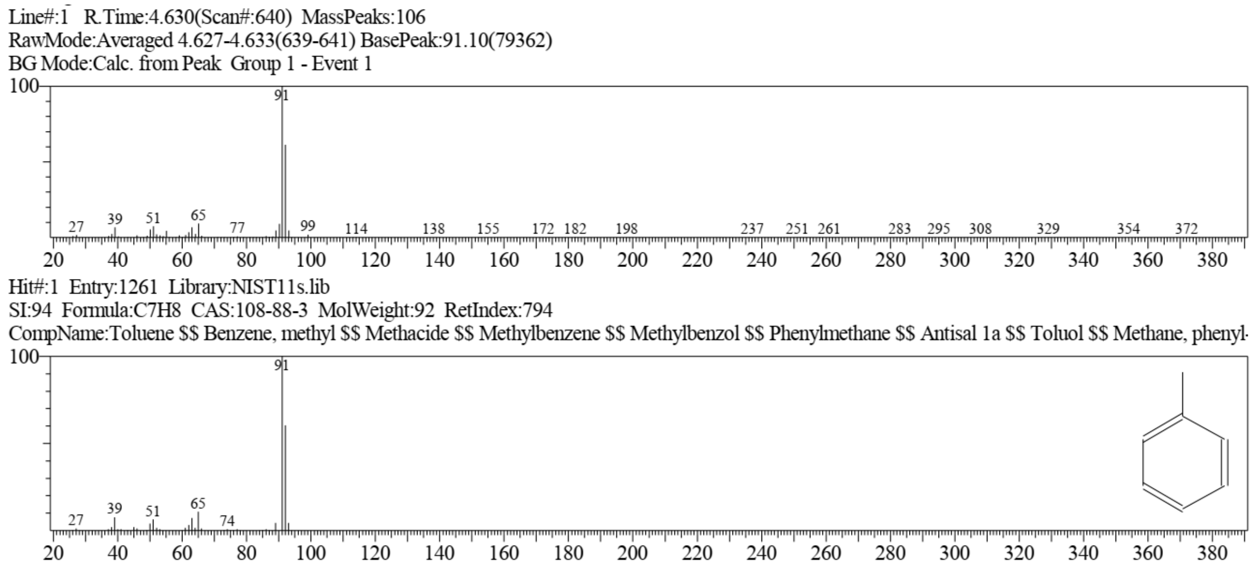

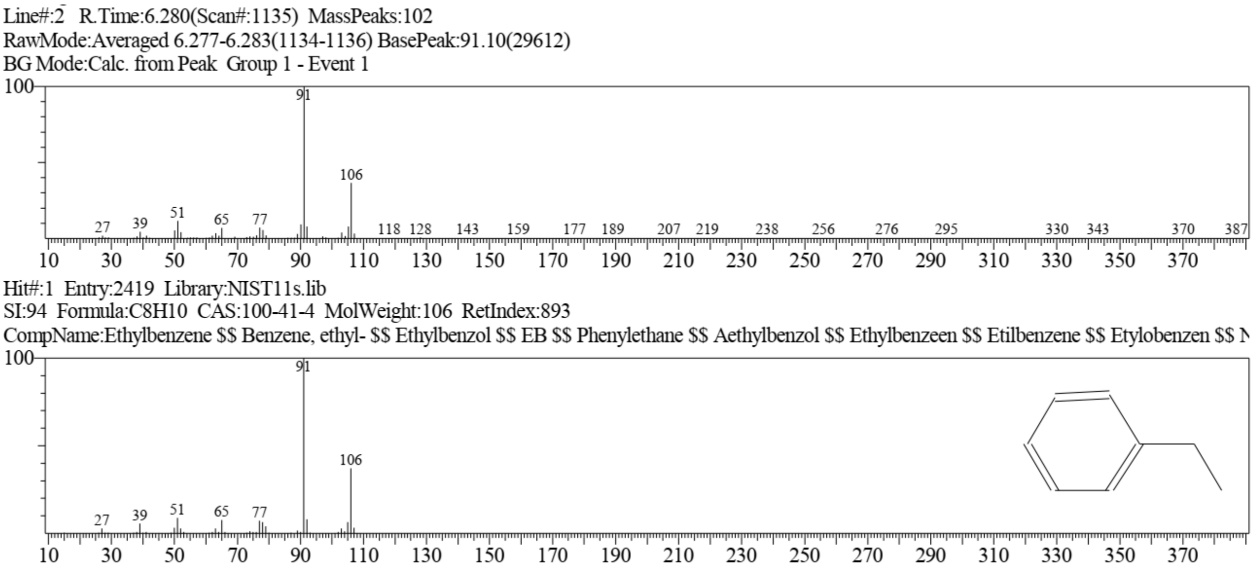

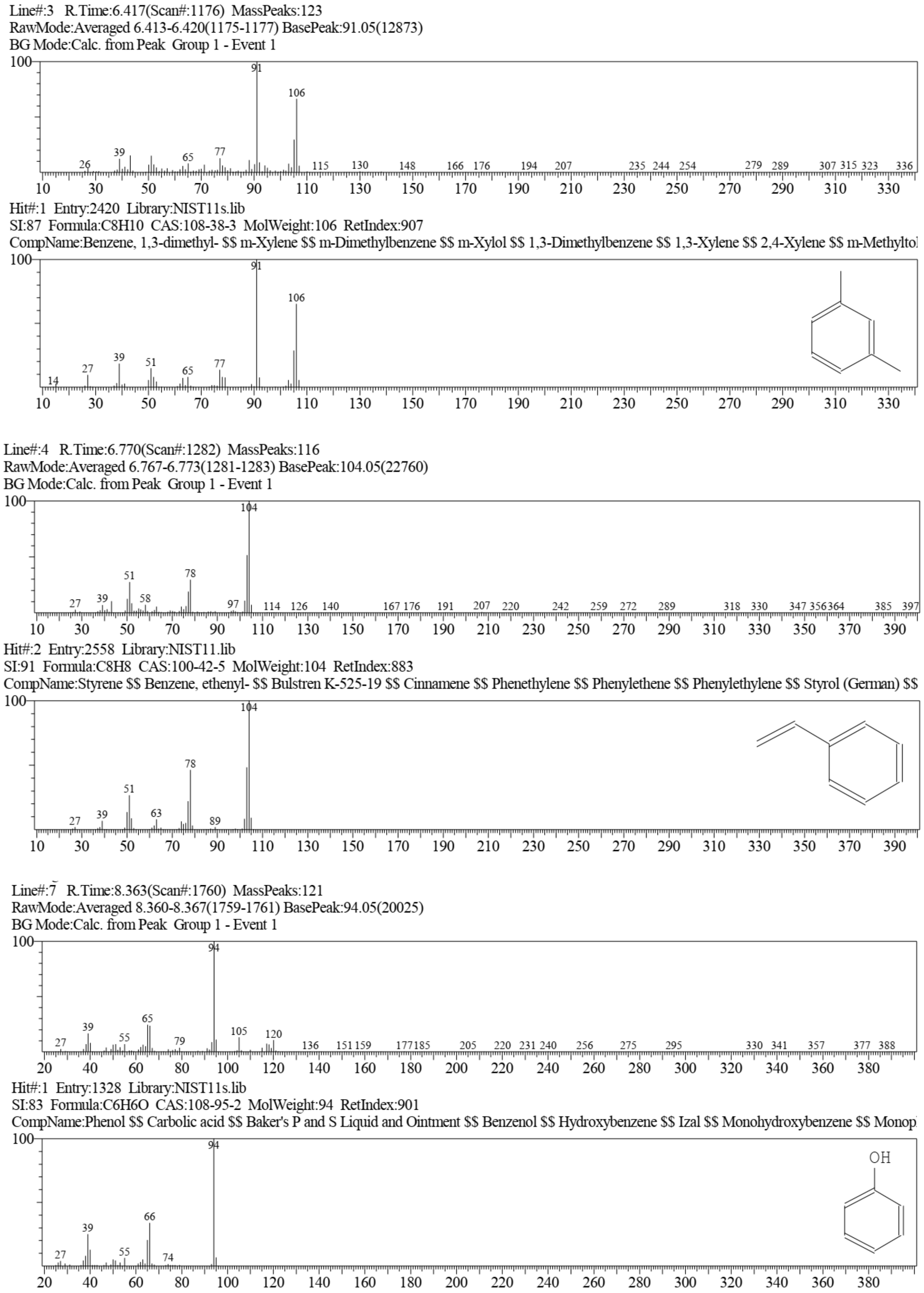
Line#:3 R.Time:6.417(Scan#:1176) MassPeaks:123
RawMode:Averaged 6.413-6.420(1175-1177) BasePeak:91.05(12873)
BG Mode:Calc. from Peak Group 1 - Event 1
Hit#:1 Entry:2420 Library:NIST11s.lib
SI:87 Formula:C8H10 CAS:108-38-3 MolWeight:106 RetIndex:907
CompName:Benzene, 1,3-dimethyl- $$ m-Xylene $$ m-Dimethylbenzene $$ m-Xylol $$ 1,3-Dimethylbenzene $$ 1,3-Xylene $$ 2,4-Xylene $$ m-Methyltol
Line#:4 R.Time:6.770(Scan#:1282) MassPeaks:116
RawMode:Averaged 6.767-6.773(1281-1283) BasePeak:104.05(22760)
BG Mode:Calc. from Peak Group 1 - Event 1
Hit#:2 Entry:2558 Library:NIST11.lib
SI:91 Formula:C8H8 CAS:100-42-5 MolWeight:104 RetIndex:883
CompName:Styrene $$ Benzene, ethenyl- $$ Bulstren K-525-19 $$ Cinnamene $$ Phenethylene $$ Phenylethene $$ Phenylethylene $$ Styrol (German) $$
Line#:7 R.Time:8.363(Scan#:1760) MassPeaks:121
RawMode:Averaged 8.360-8.367(1759-1761) BasePeak:94.05(20025)
BG Mode:Calc. from Peak Group 1 - Event 1
Hit#:1 Entry:1328 Library:NIST11s.lib
SI:83 Formula:C6H6O CAS:108-95-2 MolWeight:94 RetIndex:901
CompName:Phenol $$ Carbolic acid $$ Baker's P and S Liquid and Ointment $$ Benzenol $$ Hydroxybenzene $$ Izal $$ Monohydroxybenzene $$ Monop
OH

Line#:9 R.Time:8.723(Scan#:1868) MassPeaks:119
RawMode:Averaged 8.720-8.727(1867-1869) BasePeak:122.10(56131)
BG Mode:Calc. from Peak Group 1 - Event 1

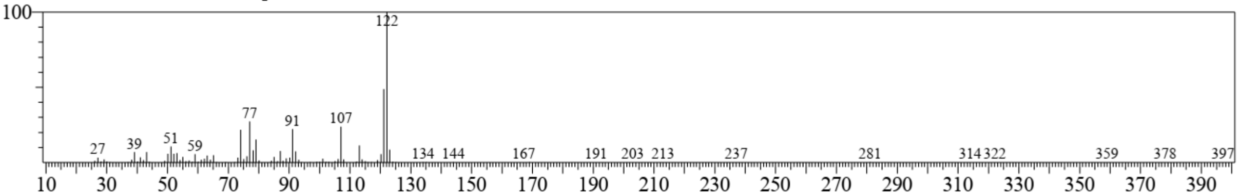

Hit#:1 Entry:5627 Library:NIST11.lib
SI:87 Formula:C8H10O CAS:104-93-8 MolWeight:122 RetIndex:983
CompName:Benzene, 1-methoxy-4-methyl- $$ Anisole, p-methyl- $$ p-Cresol methyl ether $$ p-Methoxytoluene $$ p-Methylanisole $$ p-Tolyl methyl ethe

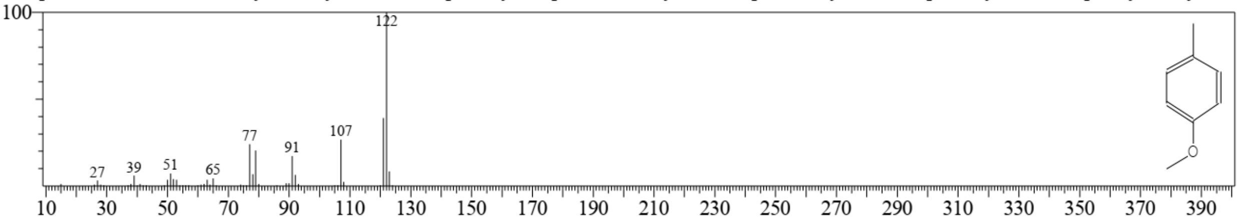

Line#:11 R.Time:9.293(Scan#:2039) MassPeaks:143
RawMode:Averaged 9.290-9.297(2038-2040) BasePeak:108.05(35109)
BG Mode:Calc. from Peak Group 1 - Event 1

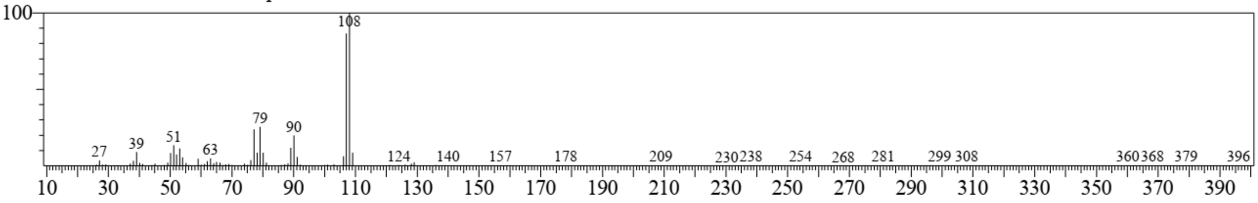

Hit#:1 Entry:2558 Library:NIST11s.lib
SI:94 Formula:C7H8O CAS:95-48-7 MolWeight:108 RetIndex:1014
CompName:Phenol, 2-methyl- $$ o-Cresol $$ o-Hydroxytoluene $$ o-Methylphenol $$ o-Methylphenylol $$ o-Oxytoluene $$ 1-Hydroxy-2-methylbenzene

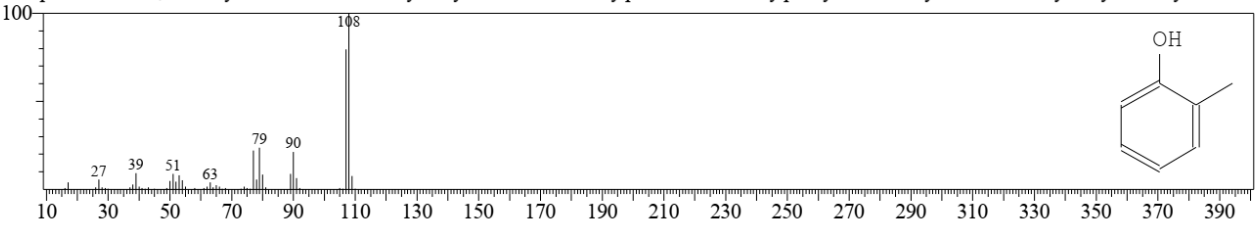

Line#:12 R.Time:9.590(Scan#:2128) MassPeaks:128
RawMode:Averaged 9.587-9.593(2127-2129) BasePeak:107.05(28425)
BG Mode:Calc. from Peak Group 1 - Event 1

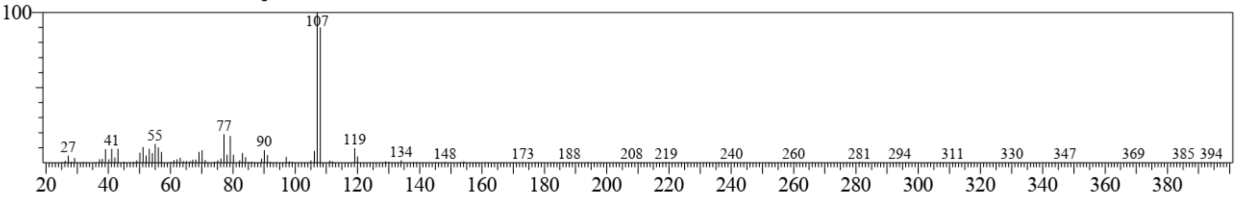

Hit#:1 Entry:2550 Library:NIST11s.lib
SI:86 Formula:C7H8O CAS:106-44-5 MolWeight:108 RetIndex:1014
CompName:p-Cresol $$ Phenol, 4-methyl- $$ p-Hydroxytoluene $$ p-Kresol $$ p-Methylhydroxybenzene $$ p-Methylphenol $$ p-Oxytoluene $$ p-Toluol

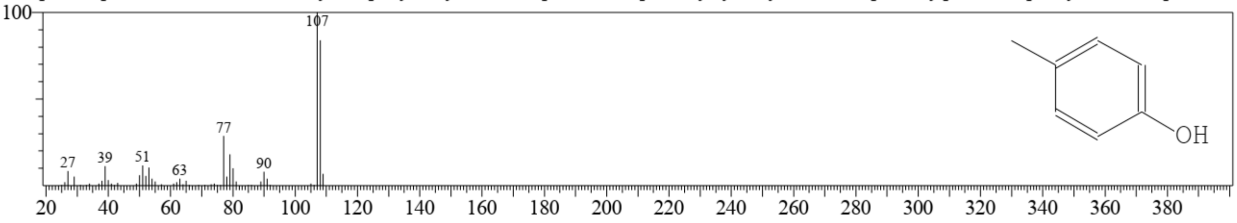

Line#:13 R.Time:9.687(Scan#:2157) MassPeaks:121
RawMode:Averaged 9.683-9.690(2156-2158) BasePeak:124.05(146275)
BG Mode:Calc. from Peak Group 1 - Event 1

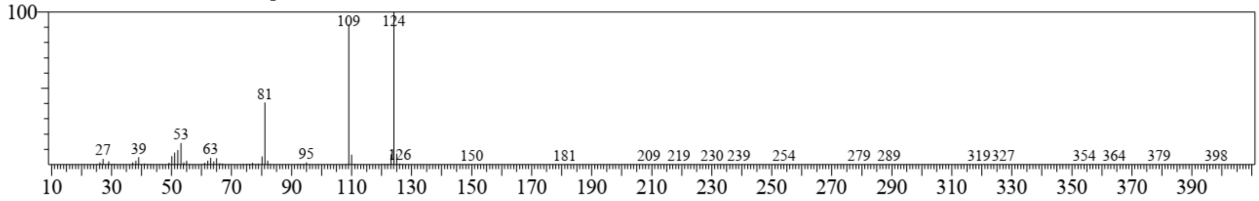

Hit#:1 Entry:4516 Library:NIST11s.lib
SI:95 Formula:C7H8O2 CAS:90-05-1 MolWeight:124 RetIndex:1090
CompName:Phenol, 2-methoxy- $$ Phenol, o-methoxy- $$ o-Guaiacol $$ o-Hydroxyanisole $$ o-Methoxyphenol $$ Anastil $$ Guaiacol $$ Guaiastil $$ Gu

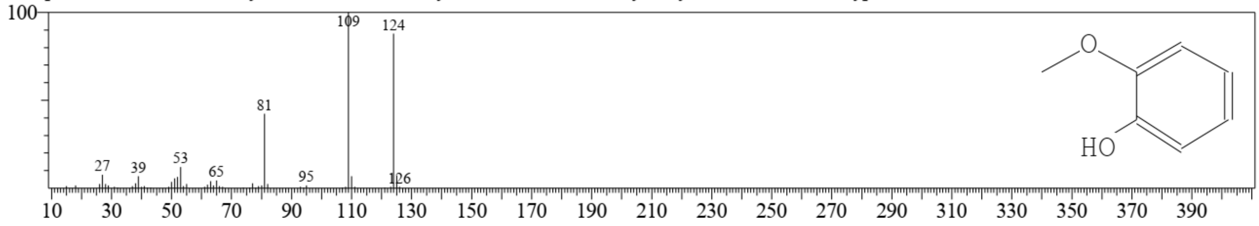

Line#:16 R.Time:10.307(Scan#:2343) MassPeaks:118
RawMode:Averaged 10.303-10.310(2342-2344) BasePeak:138.05(40187)
BG Mode:Calc. from Peak Group 1 - Event 1

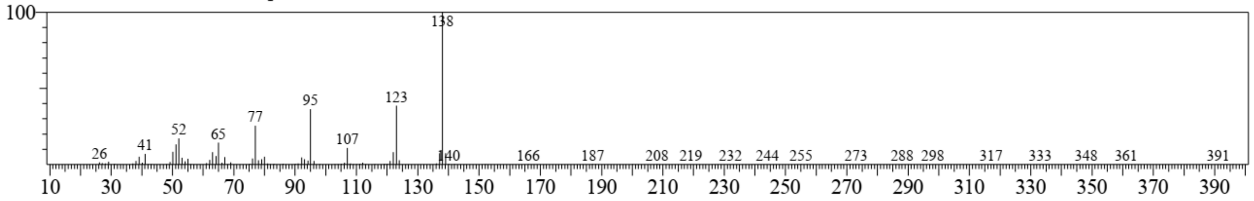

Hit#:1 Entry:6930 Library:NIST11s.lib
SI:93 Formula:C8H10O2 CAS:91-16-7 MolWeight:138 RetIndex:1059
CompName:Benzene, 1,2-dimethoxy- $$ Benzene, o-dimethoxy- $$ o-Dimethoxybenzene $$ O,O-Dimethyl catechol $$ Pyrocatechol dimethyl ether $$ Ver

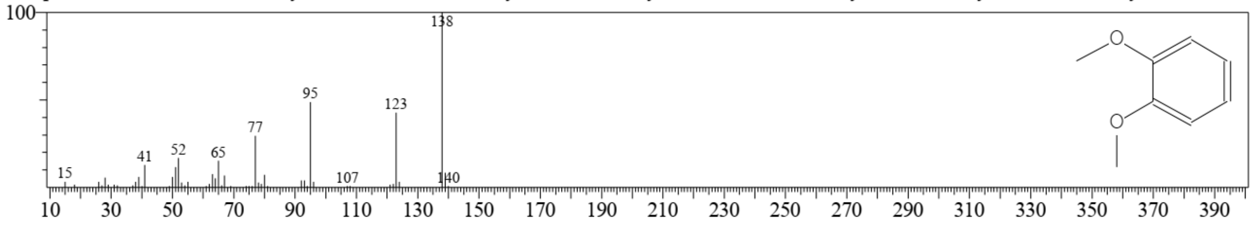

Line#:17 R.Time:10.433(Scan#:2381) MassPeaks:150
RawMode:Averaged 10.430-10.437(2380-2382) BasePeak:122.05(45403)
BG Mode:Calc. from Peak Group 1 - Event 1

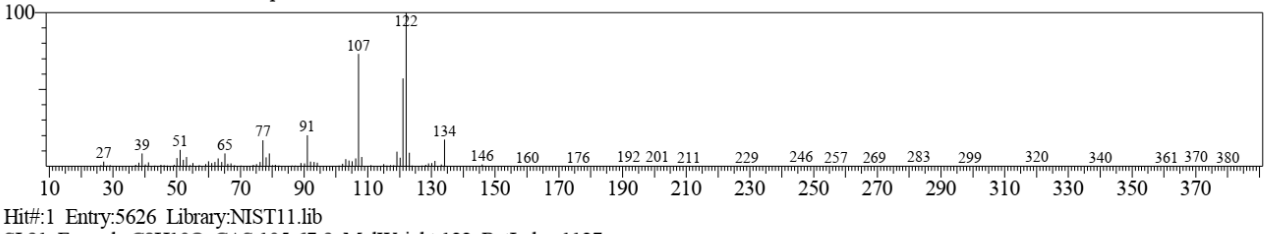

Hit#:1 Entry:5626 Library:NIST11.lib
SI:91 Formula:C8H10O CAS:105-67-9 MolWeight:122 RetIndex:1127
CompName:Phenol, 2,4-dimethyl- $$ 2,4-Xylenol $$ m-Xylenol $$ 1-Hydroxy-2,4-dimethylbenzene $$ 2,4-Dimethylphenol $$ 4-Hydroxy-1,3-dimethylben

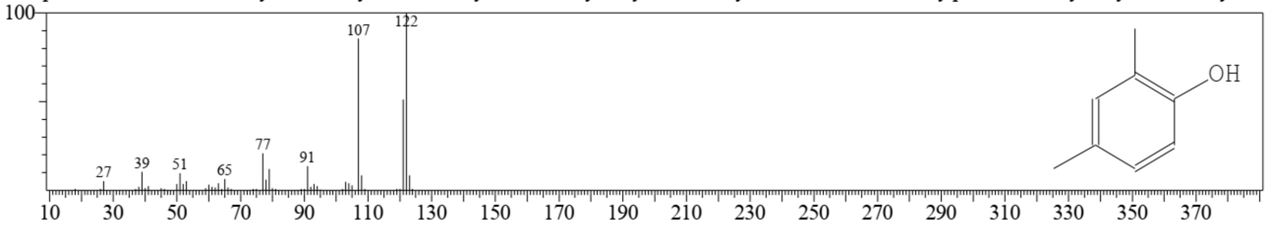

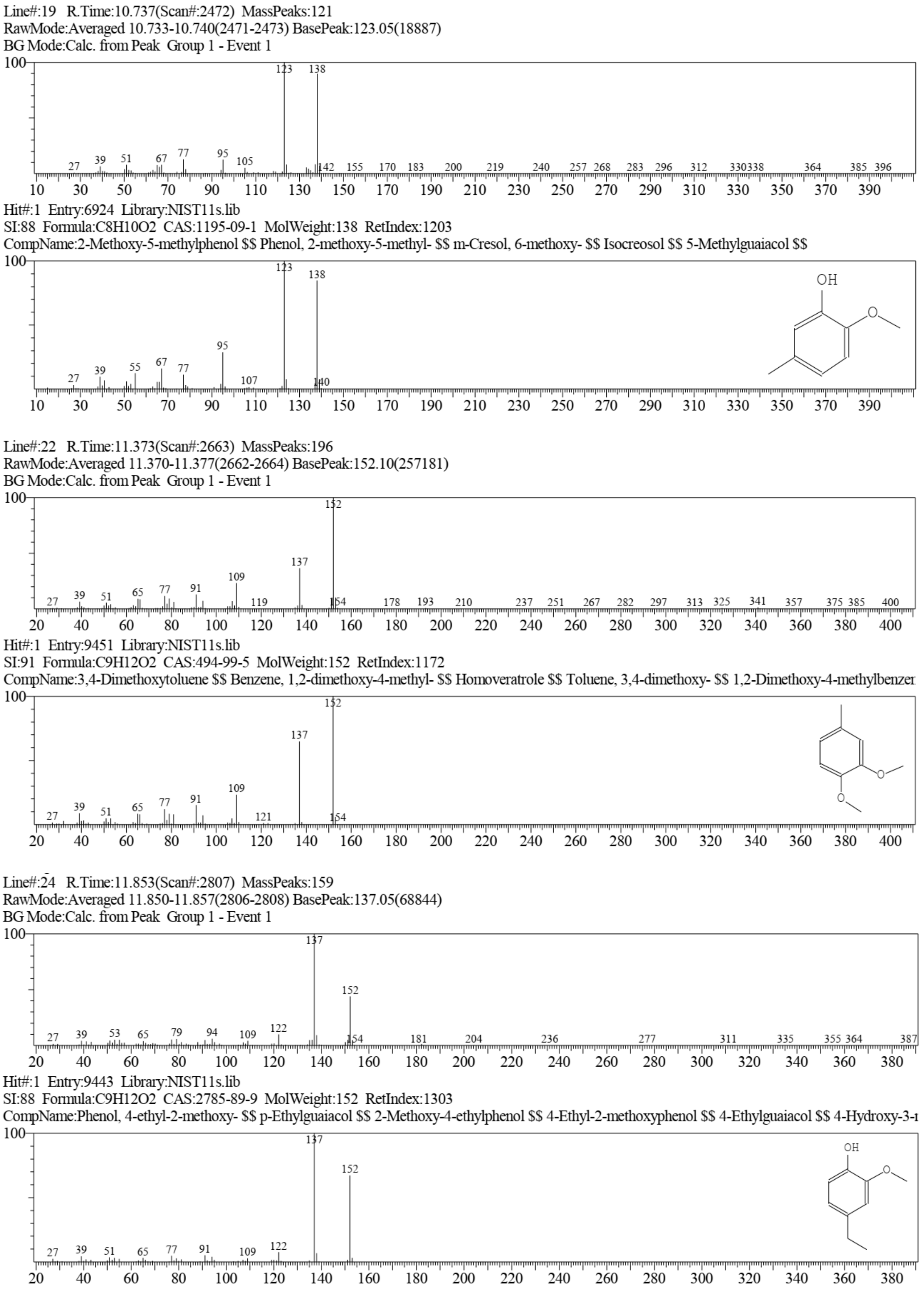
Line#:19 R.Time:10.737(Scan#:2472) MassPeaks:121
RawMode:Averaged 10.733-10.740(2471-2473) BasePeak:123.05(18887)
BG Mode:Calc. from Peak Group 1 - Event 1
Hit#:1 Entry:6924 Library:NIST11s.lib
SI:88 Formula:C8H10O2 CAS:1195-09-1 MolWeight:138 RetIndex:1203
CompName:2-Methoxy-5-methylphenol $$ Phenol, 2-methoxy-5-methyl- $$ m-Cresol, 6-methoxy- $$ Isocreosol $$ 5-Methylguaiacol $$
OH
O
Line#:22 R.Time:11.373(Scan#:2663) MassPeaks:196
RawMode:Averaged 11.370-11.377(2662-2664) BasePeak:152.10(257181)
BG Mode:Calc. from Peak Group 1 - Event 1
Hit#:1 Entry:9451 Library:NIST11s.lib
SI:91 Formula:C9H12O2 CAS:494-99-5 MolWeight:152 RetIndex:1172
CompName:3,4-Dimethoxytoluene $$ Benzene, 1,2-dimethoxy-4-methyl- $$ Homoveratrole $$ Toluene, 3,4-dimethoxy- $$ 1,2-Dimethoxy-4-methylbenzer
O
O
Line#:24 R.Time:11.853(Scan#:2807) MassPeaks:159
RawMode:Averaged 11.850-11.857(2806-2808) BasePeak:137.05(68844)
BG Mode:Calc. from Peak Group 1 - Event 1
Hit#:1 Entry:9443 Library:NIST11s.lib
SI:88 Formula:C9H12O2 CAS:2785-89-9 MolWeight:152 RetIndex:1303
CompName:Phenol, 4-ethyl-2-methoxy- $$ p-Ethylguaiacol $$ 2-Methoxy-4-ethylphenol $$ 4-Ethyl-2-methoxyphenol $$ 4-Ethylguaiacol $$ 4-Hydroxy-3-r
OH
O

Line#:25 R.Time:12.253(Scan#:2927) MassPeaks:184
RawMode:Averaged 12.250-12.257(2926-2928) BasePeak:150.10(94122)
BG Mode:Calc. from Peak Group 1 - Event 1

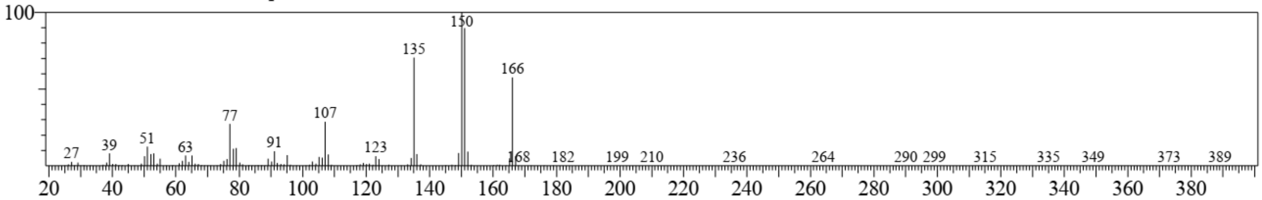

Hit#:1 Entry:23513 Library:NIST11.lib
SI:81 Formula:C10H14O2 CAS:5888-51-7 MolWeight:166 RetIndex:1271
CompName:Benzene, 4-ethyl-1,2-dimethoxy- $$ 4-Ethyl-1,2-dimethoxybenzene $$ 4-Ethyl-2-methoxyanisole $$

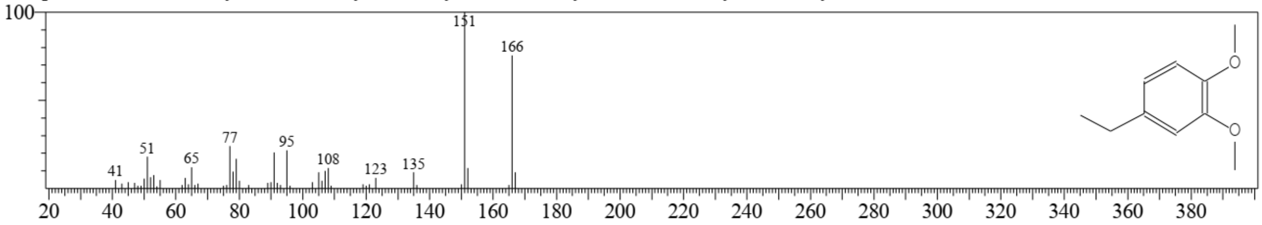

Line#:27 R.Time:12.720(Scan#:3067) MassPeaks:164
RawMode:Averaged 12.717-12.723(3066-3068) BasePeak:164.05(222765)
BG Mode:Calc. from Peak Group 1 - Event 1

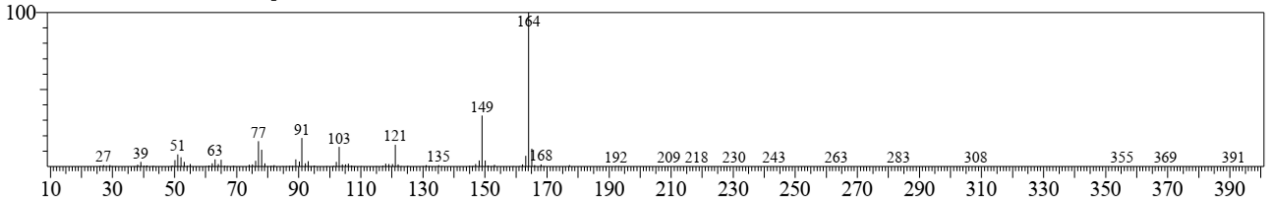

Hit#:1 Entry:22317 Library:NIST11.lib
SI:91 Formula:C10H12O2 CAS:6380-23-0 MolWeight:164 RetIndex:1261
CompName:Benzene, 4-ethenyl-1,2-dimethoxy- $$ 3,4-Dimethoxystyrene $$ 1,2-Dimethoxy-4-vinylbenzene $$ 4-Ethenyl-1,2-dimethoxybenzene $$ 4-Viny

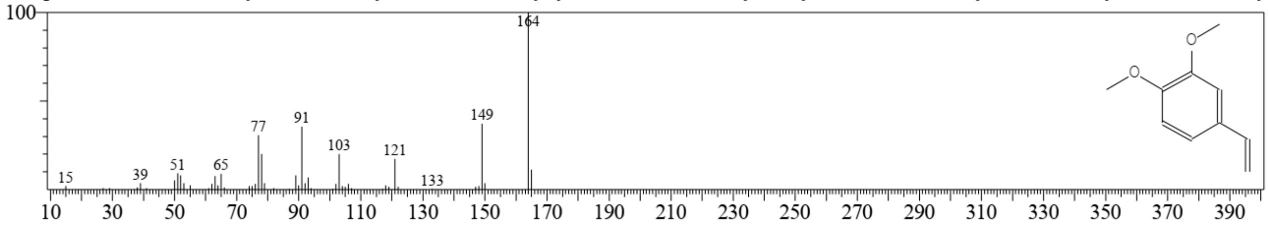

Line#:28 R.Time:12.937(Scan#:3132) MassPeaks:185
RawMode:Averaged 12.933-12.940(3131-3133) BasePeak:131.05(12449)
BG Mode:Calc. from Peak Group 1 - Event 1

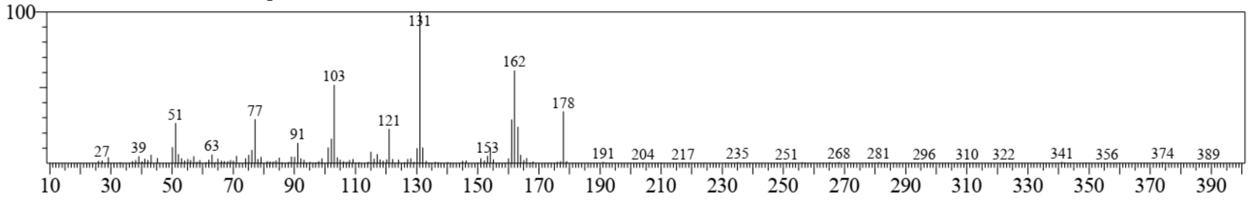

Hit#:1 Entry:21207 Library:NIST11.lib
SI:81 Formula:C10H10O2 CAS:103-26-4 MolWeight:162 RetIndex:1267
CompName:2-Propenoic acid, 3-phenyl-, methyl ester $$ Cinnamic acid, methyl ester $$ Methyl cinnamate $$ Methyl cinnamylate $$ Methyl 3-phenylprope

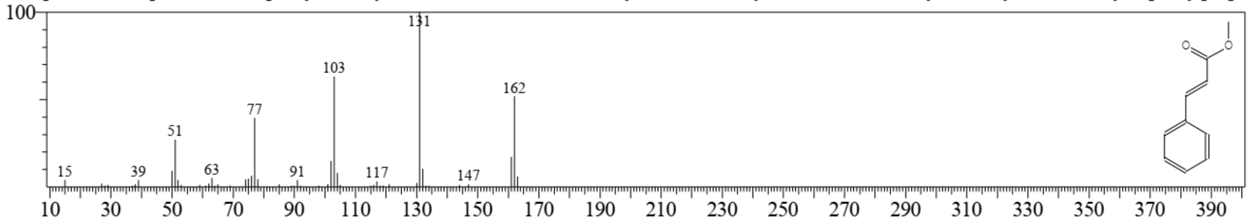

Line#:29 R.Time:13.053(Scan#:3167) MassPeaks:170
RawMode:Averaged 13.050-13.057(3166-3168) BasePeak:178.10(32703)
BG Mode:Calc. from Peak Group 1 - Event 1

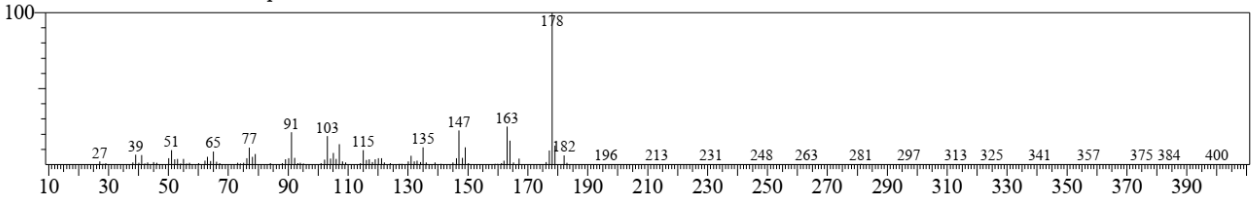

Hit#:1 Entry:14058 Library:NIST11s.lib
SI:89 Formula:C11H14O2 CAS:93-15-2 MolWeight:178 RetIndex:1361
CompName:Methyleugenol $$ Benzene, 1,2-dimethoxy-4-(2-propenyl)- $$ Benzene, 4-allyl-1,2-dimethoxy- $$ Ent 21040 $$ Eugenol methyl ether $$ Euge

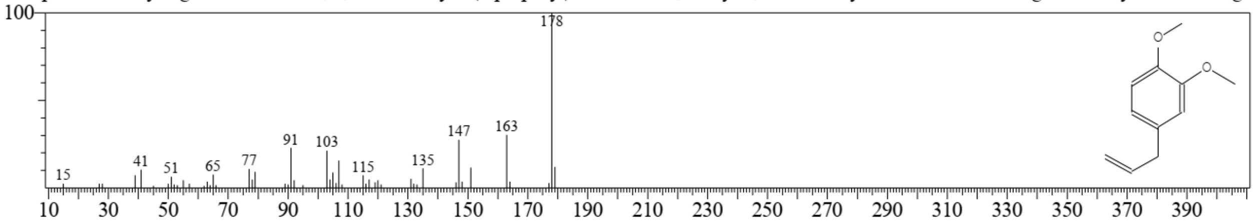

Line#:30 R.Time:13.187(Scan#:3207) MassPeaks:147
RawMode:Averaged 13.183-13.190(3206-3208) BasePeak:151.05(28045)
BG Mode:Calc. from Peak Group 1 - Event 1

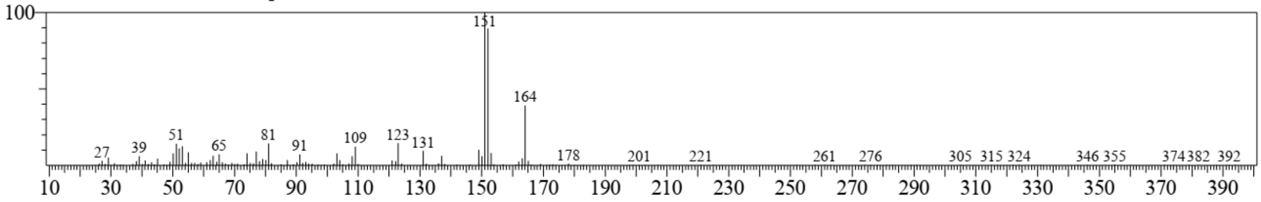

Hit#:1 Entry:16084 Library:NIST11.lib
SI:83 Formula:C8H8O3 CAS:121-33-5 MolWeight:152 RetIndex:1392
CompName:Vanillin $$ Benzaldehyde, 4-hydroxy-3-methoxy- $$ p-Hydroxy-m-methoxybenzaldehyde $$ Lioxin $$ Vanillaldehyde $$ Vanillic aldehyde $$

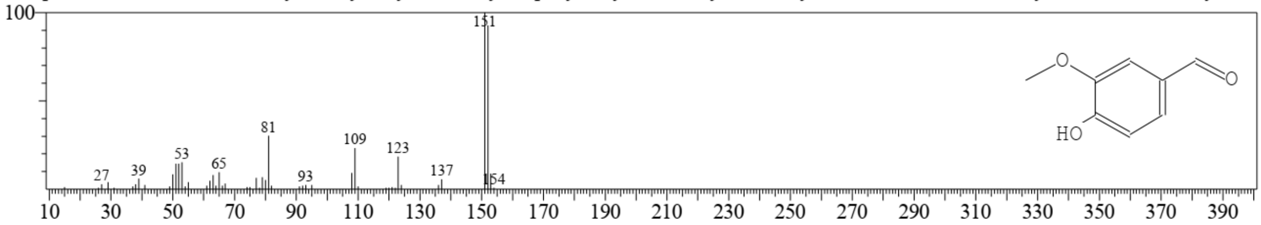

Line#:31 R.Time:13.587(Scan#:3327) MassPeaks:181
RawMode:Averaged 13.583-13.590(3326-3328) BasePeak:164.10(69935)
BG Mode:Calc. from Peak Group 1 - Event 1

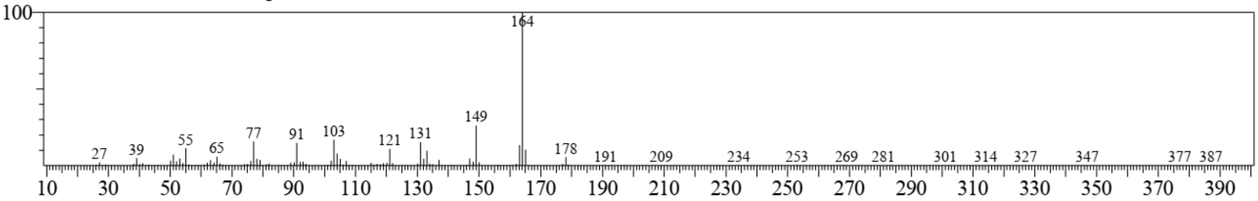

Hit#:1 Entry:11663 Library:NIST11s.lib
SI:90 Formula:C10H12O2 CAS:5932-68-3 MolWeight:164 RetIndex:1410
CompName:trans-Isoeugenol $$ Phenol, 2-methoxy-4-(1-propenyl)-, (E)- $$ Phenol, 2-methoxy-4-propenyl-, (E)- $$ (E)-Isoeugenol $$ trans-p-Propenylqua

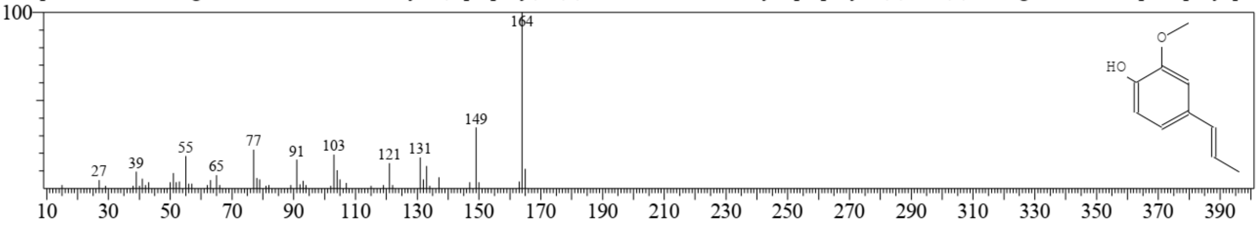

Line#:32 R.Time:13.893(Scan#:3419) MassPeaks:162
RawMode:Averaged 13.890-13.897(3418-3420) BasePeak:166.05(255623)
BG Mode:Calc. from Peak Group 1 - Event 1

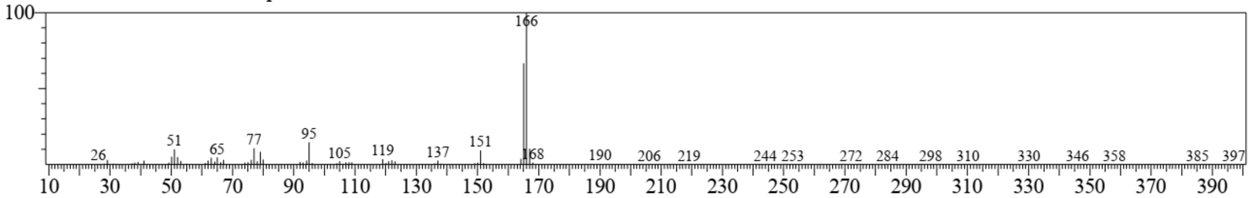

Hit#:1 Entry:12025 Library:NIST11s.lib
SI:94 Formula:C9H10O3 CAS:120-14-9 MolWeight:166 RetIndex:1360
CompName:Benzaldehyde, 3,4-dimethoxy- $$ Veratraldehyde $$ Methylvanillin $$ Protocatechualdehyde dimethyl ether $$ Protocatechuic aldehyde dimet

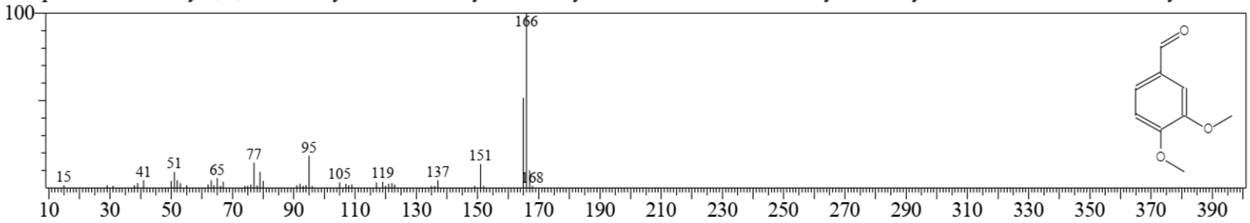

Line#:33 R.Time:13.967(Scan#:3441) MassPeaks:189
RawMode:Averaged 13.963-13.970(3440-3442) BasePeak:178.10(88829)
BG Mode:Calc. from Peak Group 1 - Event 1

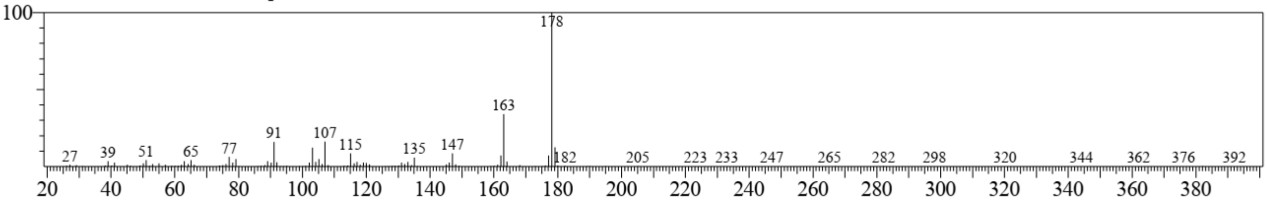

Hit#:1 Entry:14056 Library:NIST11s.lib
SI:91 Formula:C11H14O2 CAS:93-16-3 MolWeight:178 RetIndex:1379
CompName:Benzene, 1,2-dimethoxy-4-(1-propenyl)- $$ Benzene, 1,2-dimethoxy-4-propenyl- $$ Isoeugenol methyl ether $$ Isoeugenyl methyl ether $$ Iso

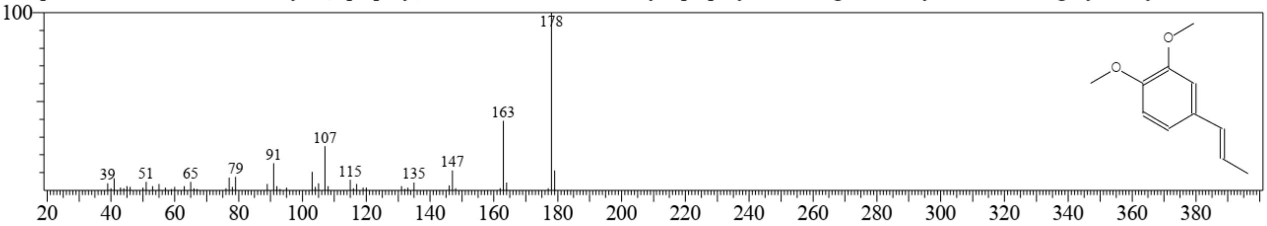

Line#:34 R.Time:14.017(Scan#:3456) MassPeaks:147
RawMode:Averaged 14.013-14.020(3455-3457) BasePeak:151.05(25756)
BG Mode:Calc. from Peak Group 1 - Event 1

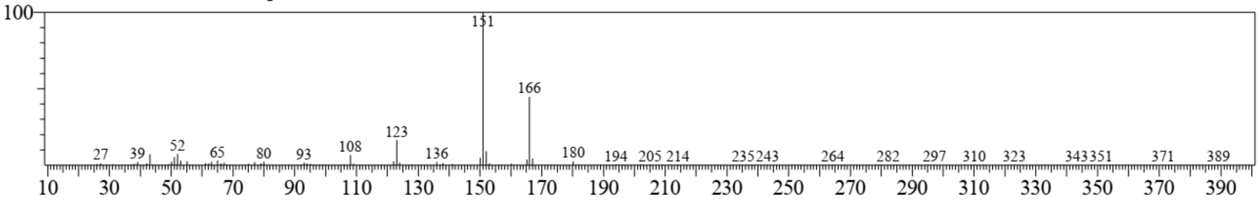

Hit#:1 Entry:12012 Library:NIST11s.lib
SI:91 Formula:C9H10O3 CAS:498-02-2 MolWeight:166 RetIndex:1439
CompName:Apocynin $$ Ethanone, 1-(4-hydroxy-3-methoxyphenyl)- $$ Acetophenone, 4'-hydroxy-3'-methoxy- $$ Acetoguaiacon $$ Acetoguaiacone $$ A

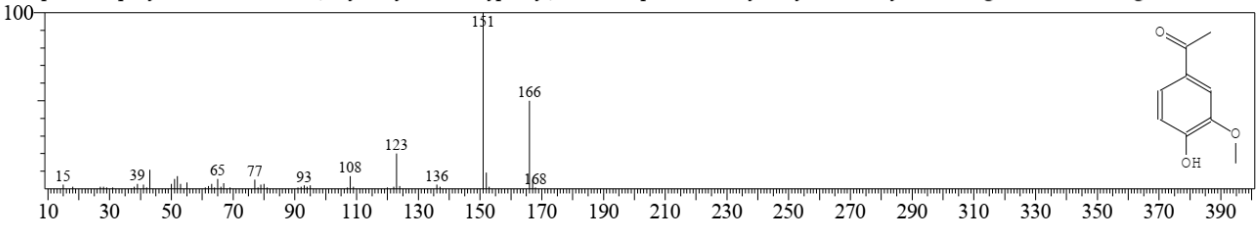

Line#:36 R.Time:14.237(Scan#:3522) MassPeaks:184
RawMode:Averaged 14.233-14.240(3521-3523) BasePeak:151.05(19133)
BG Mode:Calc. from Peak Group 1 - Event 1

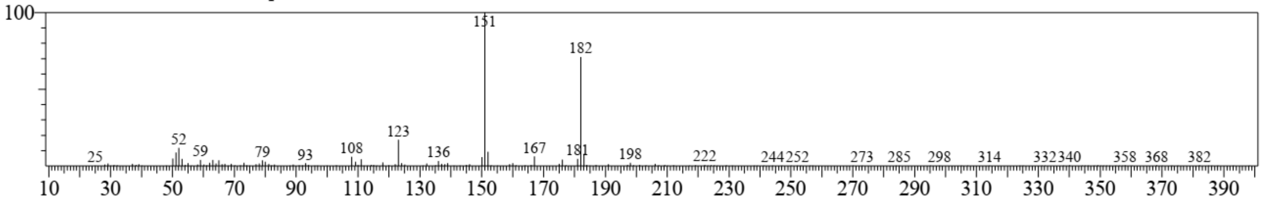

Hit#:1 Entry:32522 Library:NIST11.lib
SI:89 Formula:C9H10O4 CAS:3943-74-6 MolWeight:182 RetIndex:1470
CompName:Benzoic acid, 4-hydroxy-3-methoxy-, methyl ester $$ Vanillic acid, methyl ester $$ Methyl vanillate $$ Methyl 3-methoxy-4-hydroxybenzoate $

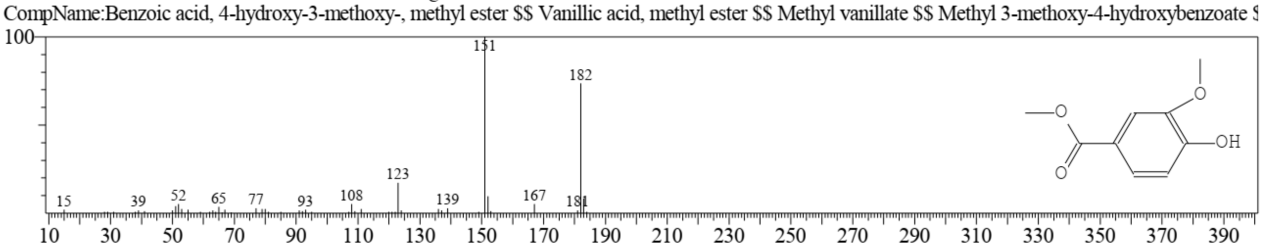

Line#:37 R.Time:14.397(Scan#:3570) MassPeaks:160
RawMode:Averaged 14.393-14.400(3569-3571) BasePeak:137.05(37361)
BG Mode:Calc. from Peak Group 1 - Event 1

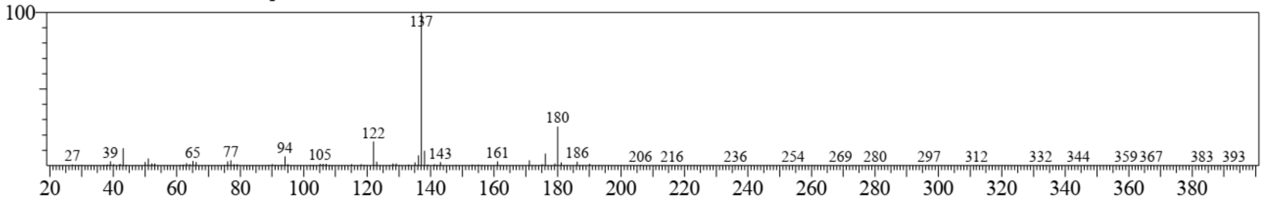

Hit#:1 Entry:14388 Library:NIST11s.lib
SI:83 Formula:C10H12O3 CAS:2503-46-0 MolWeight:180 RetIndex:1538
CompName:2-Propanone, 1-(4-hydroxy-3-methoxyphenyl)- $$ Guaiacylacetone $$ Vanillyl methyl ketone $$ 4-Hydroxy-3-methoxyphenyl acetone $$ 2-Pro

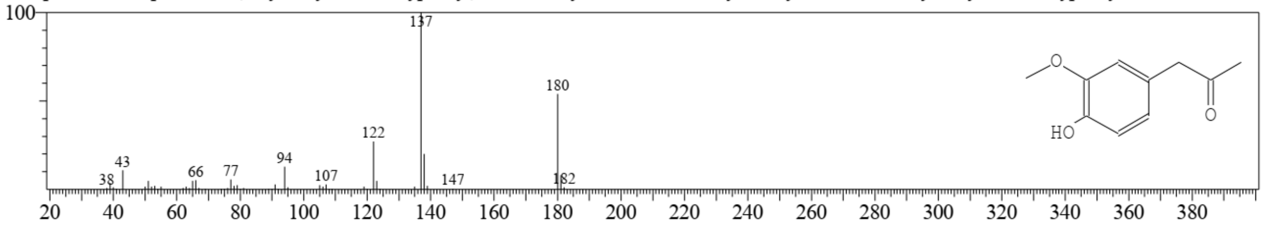

Line#:38 R.Time:14.630(Scan#:3640) MassPeaks:178
RawMode:Averaged 14.627-14.633(3639-3641) BasePeak:165.05(115918)
BG Mode:Calc. from Peak Group 1 - Event 1

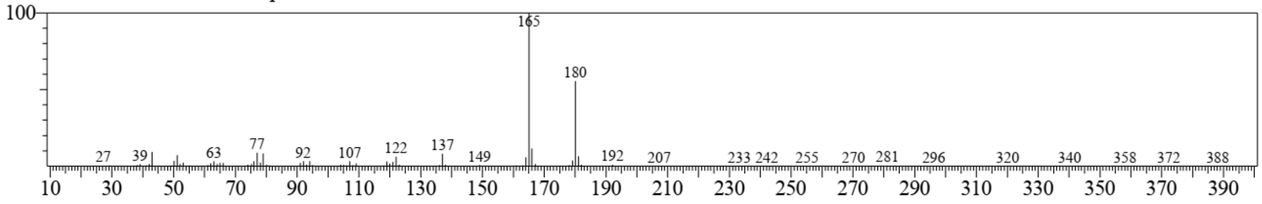

Hit#:1 Entry:14396 Library:NIST11s.lib
SI:95 Formula:C10H12O3 CAS:1131-62-0 MolWeight:180 RetIndex:1407
CompName:Ethanone, 1-(3,4-dimethoxyphenyl)- $$ Acetophenone, 3',4'-dimethoxy- $$ Acetoveratrone $$ 3,4-Dimethoxyphenyl methyl ketone $$ 3',4'-Din

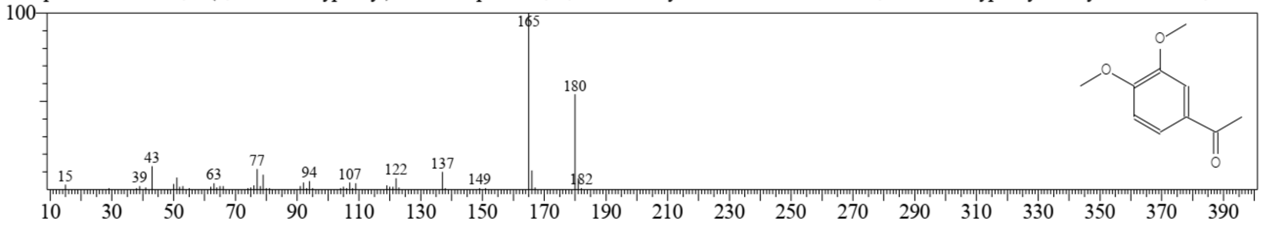

Line#:39 R.Time:14.743(Scan#:3674) MassPeaks:161
RawMode:Averaged 14.740-14.747(3673-3675) BasePeak:151.05(73959)
BG Mode:Calc. from Peak Group 1 - Event 1

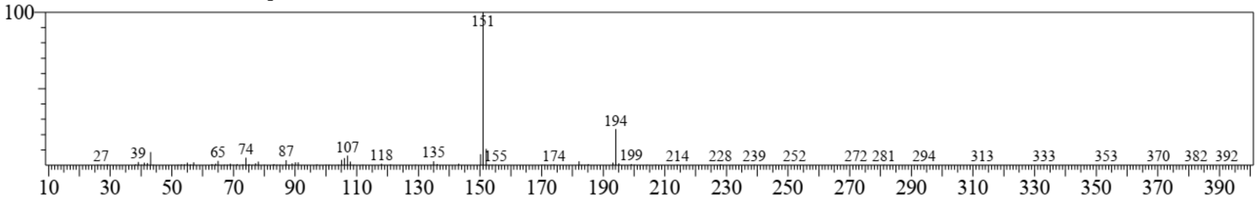

Hit#:1 Entry:16535 Library:NIST11s.lib
SI:85 Formula:C11H14O3 CAS:776-99-8 MolWeight:194 RetIndex:1507
CompName:3,4-Dimethoxyphenylacetone $$ Veratryl acetone $$ 2-Propanone, 1-(3,4-dimethoxyphenyl)- $$ 3,4-Dimethoxybenzyl methyl ketone $$ 1-(3,4-

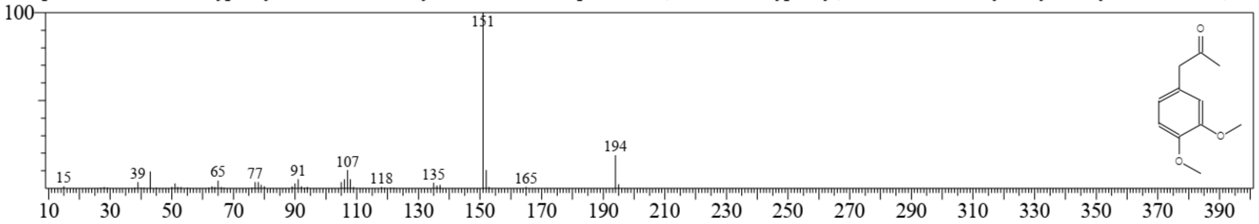

Line#:40 R.Time:14.827(Scan#:3699) MassPeaks:199
RawMode:Averaged 14.823-14.830(3698-3700) BasePeak:196.05(122464)
BG Mode:Calc. from Peak Group 1 - Event 1

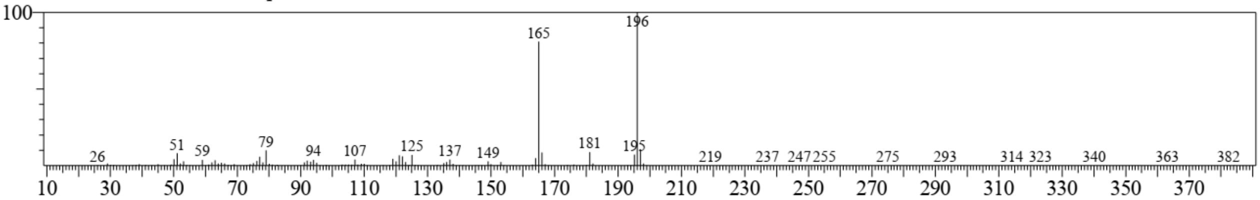

Hit#:1 Entry:41219 Library:NIST11.lib
SI:94 Formula:C10H12O4 CAS:2150-38-1 MolWeight:196 RetIndex:1439
CompName:Benzoic acid, 3,4-dimethoxy-, methyl ester $$ Veratric acid, methyl ester $$ Methyl veratrate $$ Methyl 3,4-dimethoxybenzoate $$ 3,4-Dimeth

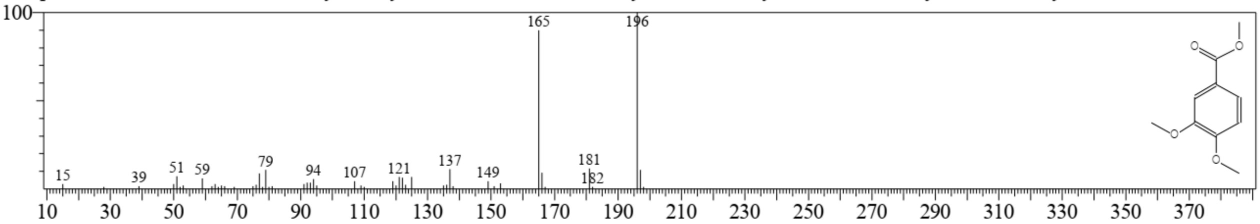

Line#:43 R.Time:15.217(Scan#:3816) MassPeaks:199
RawMode:Averaged 15.213-15.220(3815-3817) BasePeak:194.10(41000)
BG Mode:Calc. from Peak Group 1 - Event 1

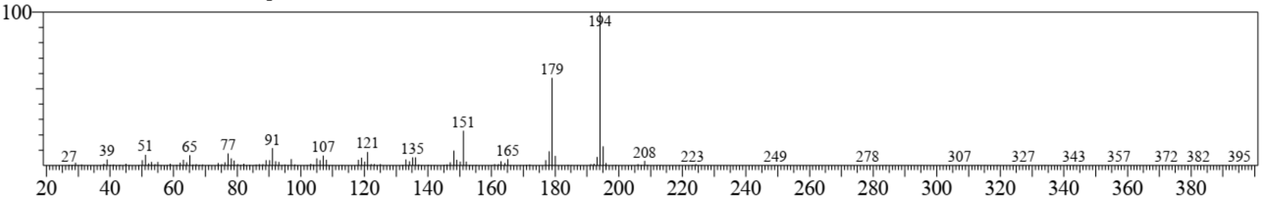

Hit#:1 Entry:39984 Library:NIST11.lib
SI:85 Formula:C11H14O3 CAS:0-00-0 MolWeight:194 RetIndex:1455
CompName:1,2-Dimethoxy-4-(2-methoxyethenyl)benzene

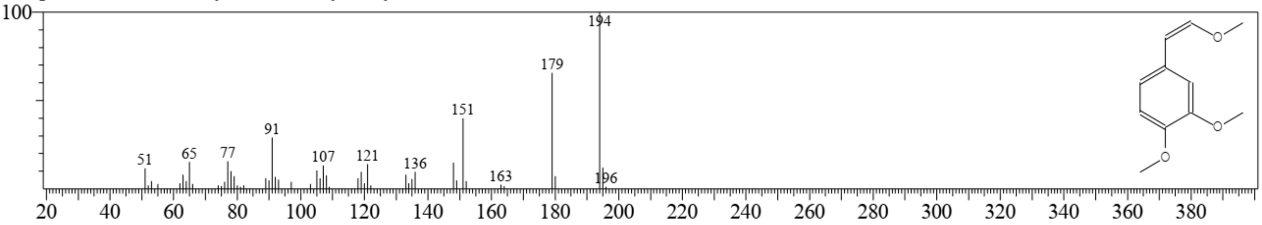

Line#:46 R.Time:15.500(Scan#:3901) MassPeaks:195
RawMode:Averaged 15.497-15.503(3900-3902) BasePeak:208.10(43419)
BG Mode:Calc. from Peak Group 1 - Event 1

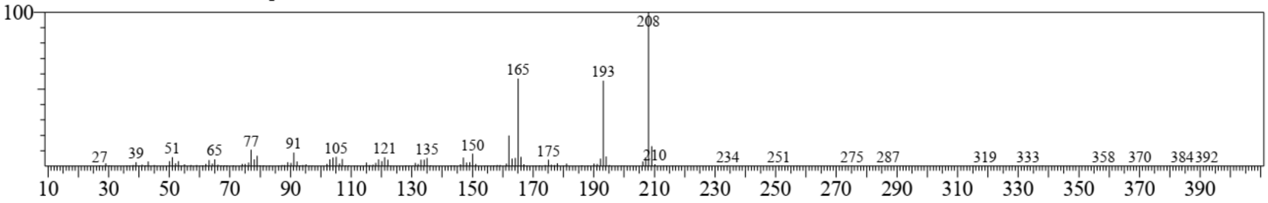

Hit#:1 Entry:49347 Library:NIST11.lib
SI:85 Formula:C12H16O3 CAS:5273-86-9 MolWeight:208 RetIndex:1568
CompName:.beta.-Asarone $$ Benzene, 1,2,4-trimethoxy-5-(1-propenyl)-, (Z)- $$ cis-Asarone $$ cis-.beta.-Asarone $$ (Z)-Asarone $$ Benzene, 1,2,4-trim

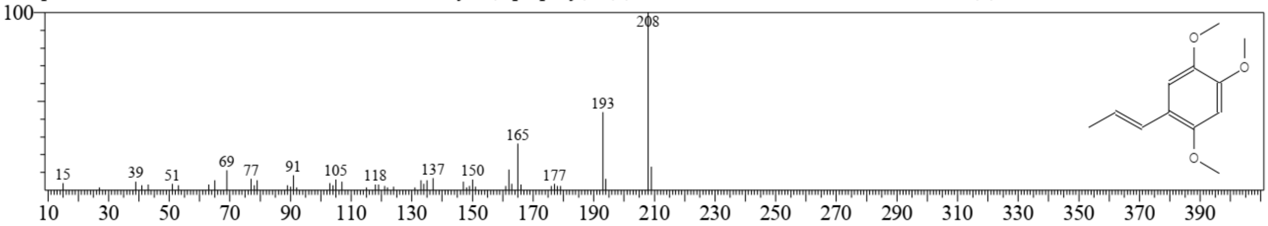

Line#:47 R.Time:15.643(Scan#:3944) MassPeaks:206
RawMode:Averaged 15.640-15.647(3943-3945) BasePeak:151.05(55268)
BG Mode:Calc. from Peak Group 1 - Event 1

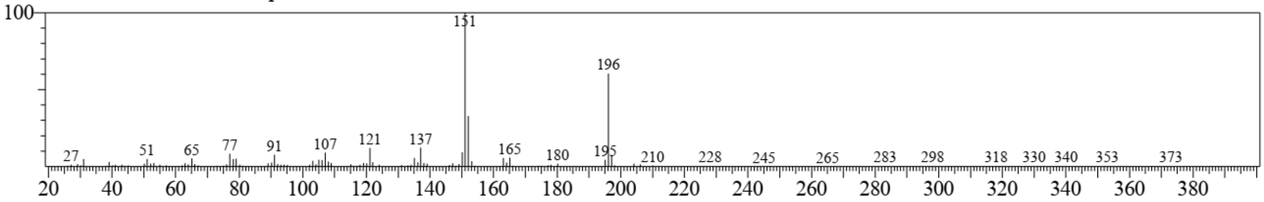

Hit#:1 Entry:41364 Library:NIST11.lib
SI:93 Formula:C11H16O3 CAS:3929-47-3 MolWeight:196 RetIndex:1613
CompName:3-(3,4-Dimethoxyphenyl)-1-propanol $$ Benzenepropanol, 3,4-dimethoxy- $$ 1-Propanol, 3-(3,4-dimethoxyphenyl) $$

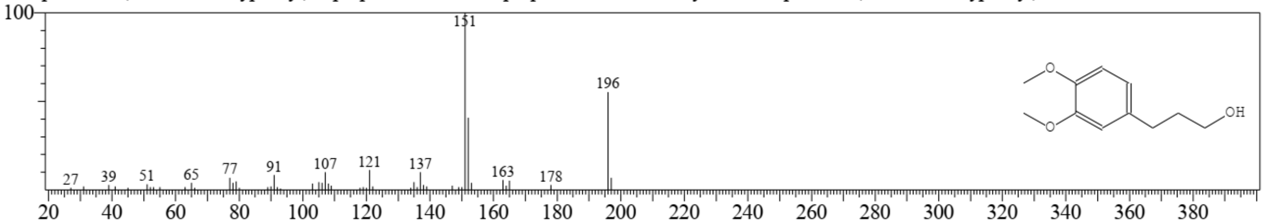

Line#:49 R.Time:15.880(Scan#:4015) MassPeaks:180
RawMode:Averaged 15.877-15.883(4014-4016) BasePeak:151.05(19948)
BG Mode:Calc. from Peak Group 1 - Event 1

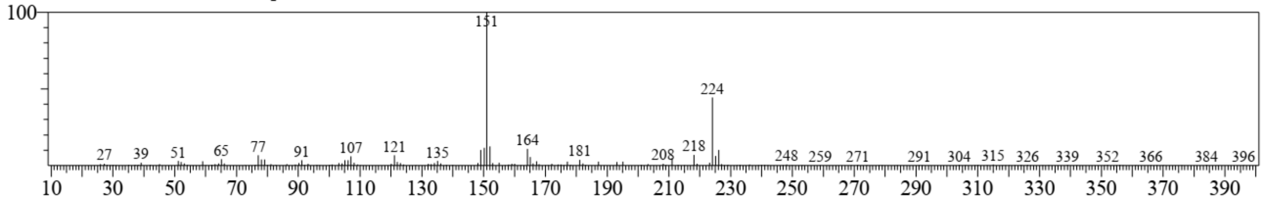

Hit#:1 Entry:60545 Library:NIST11.lib
SI:86 Formula:C12H16O4 CAS:27798-73-8 MolWeight:224 RetIndex:1637
CompName:Benzenepropanoic acid, 3,4-dimethoxy-, methyl ester $$ Hydrocinnamic acid, 3,4-dimethoxy-, methyl ester $$ Methyl 3-(3,4-dimethoxyphenyl)

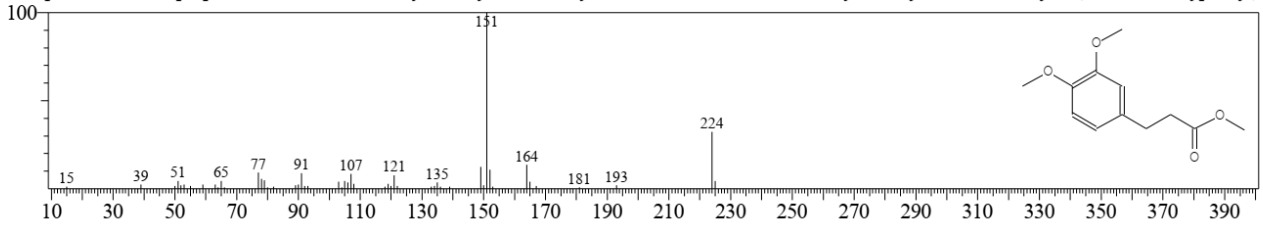

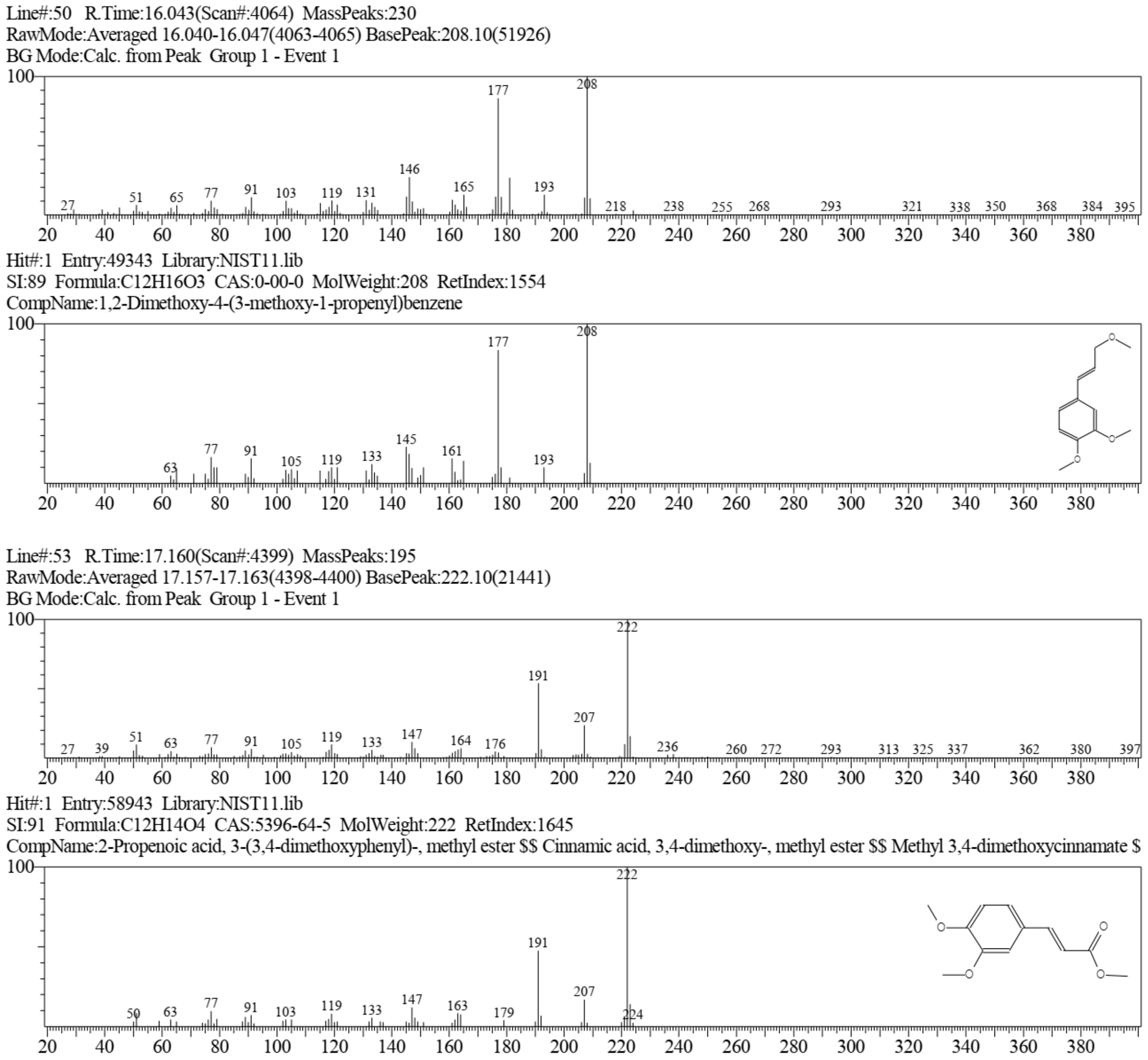

Figure S1. GC/MS library hits of lignin extracted using ChCl/EG/*p*-TsOH.
